# Supplementary material for: Distance learning in higher education during COVID-19: The role of basic psychological needs and intrinsic motivation for persistence and procrastination–a multi-country study
Source: PLoS One. 2021 Oct 6;16(10):e0257346. doi: 10.1371/journal.pone.0257346 (PMC8494346; doi:10.1371/journal.pone.0257346)
Supplement: S1 File — (DOCX) [file pone.0257346.s001.docx]

**Supplementary Online Materials**

| **Table S1**  *Details on data collection and specific circumstances during data collection for each country* | | | | | |
| --- | --- | --- | --- | --- | --- |
| Country | Time Period of Data Collection | Tool for Data collection | Recruitment | When did universities close? | Did universities remain closed during data collection? If not, what changes occurred? |
| Albania | April 25^th^ -  May 15^th^ 2020 | Google Forms | E-mail to students Google Classroom  WhatsApp  Viber | March 9^th^, 2020. | Yes |
| Austria | April 7^th^ -  April 24^th^, 2020 | Unipark | Link to the survey on the homepage of the Federal Ministry of Education, Science and Research and the homepage of the University of Vienna. Promotion via student representatives and the University Conference of the University of Vienna as well as public media. | March 16^th^, 2020 | Yes |
| China | August 5^th^ - August 20^th^ 2020 | Wenjuanxing | Academic group in social media WeChat | January 14^th^, 2020 | Yes |
| Croatia | May 1^st^ -  June 17^th^ 2020 | Google Forms | E-mail to students | March 16^th^, 2020 | Yes, mostly. As of June 15^th^, 2020, nursing students were allowed to attend practical lessons on-site. |
| Estonia^a^ | May 11^th^ -  June 25^th^ 2020 | Lime Survey | E-mail to universities Social media Hostels Word of mouth (personal contacts) | March 14^th^, 2020 | Yes, mostly. As of May 18^th^, 2020 assessment and exams could carried on-site if necessary. |
| Finland | April 29^th^ -  June 9^th^ 2020 | Qualtrics | Faculty e-mail lists University of Helsinki’s social media channels | March 18^th^ 2020 | Yes |
| Germany | May 5^th^ to June 11^th^, 2020 | Unipark | E-mail to students Published in a mailing list for ongoing data collections | March 20^th^, 2020 | Yes |
| Iceland | April 27^th^ -  June 4^th^, 2020 | Google Forms | E-mails to universities Social media | March 16^th^, 2020 | First steps out of lockdown were taken as of May 4^th^, 2020, however lecture period was over by then (most exams took place at the end of April, some in May). |
| Japan | Jun 19^th^ -  Aug 14^th^, 2020 | Google Forms | E-mail to students Colleagues | April 7^th^, 2020 at the latest | Yes |
| Kosovo | May 10^th^ -  May 28^th^, 2020 | Google Forms | University e-mails Social media University professors & students’ representatives of different departments shared the link Contacted private university representatives | March 11^th^, 2020 | Yes |
| Lithuania | May 2^nd^ -  June 10^th^, 2020 | Google Forms | Contact with representatives at the universities (vice-deans responsible for studies at the faculties) and representatives of students’ associations at the universities/colleges.  Contact with colleagues from other universities with the plea to share the link to share with their students Emails to own students with the request to share the link | March 16^th^, 2020 | Yes |
| Malta | May 12^th^ -  May 19^th^, 2020 | Google Forms | E-Mails to all students of EQF Level 6 study programs via administration of respective universities. | March 13^th^, 2020 | Yes |
| North Macedonia | April 26^th^ -  June 18^th^, 2020 | Google Forms | Social media (Facebook) E-mails to universities  E-mails to students | March 11^th^, 2020 | Yes |
| Poland | May 18^th^ -  June 16^th^, 2020 | Microsoft Forms | E-mails to universities (i.e., Contact with colleagues from other universities who distributed the link to their students via university's online learning platform)  E-mails to students | March 11^th^, 2020 | As of June 6^th^, 2020, laboratory classes in small groups were allowed on-site. Exams with physical presence were provided upon specific request of students (ex. lack of internet). |
| Romania | May 5^th^ -  June 1^st^,2020 | Google Forms | Social media Zoom  Skype E-mails to students | March 13^th^, 2020 | Yes |
| Sweden | May 19^th^ -  June 10^th^, 2020 | Google Forms | E-mails to universities  E-mails to student unions | March 17^th^, 2020 | Mostly. In some study programs tasks could be carried out as on-site if necessary. |
| USA | May 18^th^ -  June 1^st^, 2020 | Qualtrics | Social media | June 6th, 2020 | Yes |
| *Note.* ^a^ In Estonia, only international exchange students were surveyed. | | | | | |

| **Table S2**  *Descriptive Statistics, Correlations, and Scale Reliabilities for Albania* | | | | | | | | | | | |
| --- | --- | --- | --- | --- | --- | --- | --- | --- | --- | --- | --- |
|  | *M* | *SD* | 1 | 2 | 3 | 4 | 5 | 6 | 7 | 8 | α |
| 1 Gender | 1.19 | 0.39 | 1.00 |  |  |  |  |  |  |  |  |
| 2 Age | 21.53 | 4.33 | -.09 | 1.00 |  |  |  |  |  |  |  |
| 3 Competence | 3.25 | 1.20 | -.14^**^ | .23^**^ | 1.00 |  |  |  |  |  | .91 |
| 4 Autonomy | 3.39 | 1.24 | -.15^**^ | .16^**^ | .83^**^ | 1.00 |  |  |  |  | .86 |
| 5 Social relatedness | 3.61 | 1.03 | -.15^**^ | .14^**^ | .60^**^ | .59^**^ | 1.00 |  |  |  | .77 |
| 6 Intrinsic motivation | 3.13 | 1.16 | -.14^**^ | .19^**^ | .81^**^ | .74^**^ | .59^**^ | 1.00 |  |  | .85 |
| 7 Procrastination | 3.07 | 1.25 | .05 | -.03 | -.49^**^ | -.49^**^ | -.31^**^ | -.49^**^ | 1.00 |  | .83 |
| 8 Persistence | 3.46 | 1.15 | -.12^*^ | .08 | .61^**^ | .60^**^ | .48^**^ | .59^**^ | -.49^**^ | 1.00 | .84 |
| *Note:* Gender was coded as 1 = female, 2 = male, 3 = diverse.  *p < .05. **p < .01. ***p < .001 | | | | | | | | | | | |

| **Table S3** *Descriptive Statistics, Correlations, and Scale Reliabilities for Austria* | | | | | | | | | | | |
| --- | --- | --- | --- | --- | --- | --- | --- | --- | --- | --- | --- |
|  | *M* | *SD* | 1 | 2 | 3 | 4 | 5 | 6 | 7 | 8 | α |
| 1 Gender | 1.31 | 0.47 | 1.00 |  |  |  |  |  |  |  |  |
| 2 Age | 25.02 | 6.90 | .10^**^ | 1.00 |  |  |  |  |  |  |  |
| 3 Competence | 3.27 | 1.00 | .02 | .06^**^ | 1.00 |  |  |  |  |  | .79 |
| 4 Autonomy | 2.96 | 1.12 | .01 | .04^**^ | .61^**^ | 1.00 |  |  |  |  | .75 |
| 5 Social relatedness | 3.15 | 0.95 | -.09^**^ | -.06^**^ | .28^**^ | .22^**^ | 1.00 |  |  |  | .68 |
| 6 Intrisic motivation | 2.77 | 1.14 | .01 | .05^**^ | .63^**^ | .60^**^ | .24^**^ | 1.00 |  |  | .92 |
| 7 Procrastination | 2.95 | 1.20 | .01 | -.04^**^ | -.40^**^ | -.27^**^ | -.14^**^ | -.38^**^ | 1.00 |  | .89 |
| 8 Persistence | 3.30 | 0.95 | .05^**^ | .04^**^ | .35^**^ | .29^**^ | .15^**^ | .33^**^ | -.53^**^ | 1.00 | .79 |
| *Note:* Gender was coded as 1 = female, 2 = male, 3 = diverse.  *p < .05. **p < .01. ***p < .001 | | | | | | | | | | | |

| **Table S4** *Descriptive Statistics, Correlations, and Scale Reliabilities for China* | | | | | | | | | | | |
| --- | --- | --- | --- | --- | --- | --- | --- | --- | --- | --- | --- |
|  | *M* | *SD* | 1 | 2 | 3 | 4 | 5 | 6 | 7 | 8 | α |
| 1 Gender | 1.20 | 0.40 | 1.00 |  |  |  |  |  |  |  |  |
| 2 Age | 22.88 | 4.44 | -.16^**^ | 1.00 |  |  |  |  |  |  |  |
| 3 Competence | 3.52 | 0.60 | .11^*^ | -.03 | 1.00 |  |  |  |  |  | .74 |
| 4 Autonomy | 3.50 | 0.70 | .05 | .14^**^ | .72^**^ | 1.00 |  |  |  |  | .81 |
| 5 Social relatedness | 3.83 | 0.66 | .03 | -.02 | .55^**^ | .46^**^ | 1.00 |  |  |  | .83 |
| 6 Intrinsic motivation | 3.45 | 0.71 | .03 | .08 | .59^**^ | .53^**^ | .55^**^ | 1.00 |  |  | .91 |
| 7 Procrastination | 3.21 | 0.84 | .02 | .02 | -.06 | -.12^*^ | -.03 | -.05 | 1.00 |  | .89 |
| 8 Persistence | 3.35 | 0.70 | .11^*^ | .02 | .53^**^ | .57^**^ | .40^**^ | .51^**^ | -.20^**^ | 1.00 | .88 |
| *Note:* Gender was coded as 1 = female, 2 = male, 3 = diverse.  *p < .05. **p < .01. ***p < .001 | | | | | | | | | | | |

| **Table S5** *Descriptive Statistics, Correlations, and Scale Reliabilities for Croatia* | | | | | | | | | | | |
| --- | --- | --- | --- | --- | --- | --- | --- | --- | --- | --- | --- |
|  | *M* | *SD* | 1 | 2 | 3 | 4 | 5 | 6 | 7 | 8 | α |
| 1 Gender | 1.30 | 0.46 | 1.00 |  |  |  |  |  |  |  |  |
| 2 Age | 25.75 | 7.00 | -.22** | 1.00 |  |  |  |  |  |  |  |
| 3 Competence | 3.55 | 0.88 | -.01 | -.04 | 1.00 |  |  |  |  |  | .88 |
| 4 Autonomy | 3.74 | 0.88 | .04 | -.09 | .77^**^ | 1.00 |  |  |  |  | .85 |
| 5 Social relatedness | 3.59 | 0.83 | -.10 | -.02 | .45^**^ | .39^**^ | 1.00 |  |  |  | .68 |
| 6 Intrinsic motivation | 3.16 | 1.00 | -.05 | .06 | .58^**^ | .49^**^ | .46^**^ | 1.00 |  |  | .88 |
| 7 Procrastination | 3.21 | 1.09 | .06 | -.05 | -.37^**^ | -.28^**^ | -.18^**^ | -.39^**^ | 1.00 |  | .86 |
| 8 Persistence | 3.50 | 0.84 | .01 | -.02 | .56^**^ | .48^**^ | .36^**^ | .38^**^ | -.37^**^ | 1.00 | .80 |
| *Note:* Gender was coded as 1 = female, 2 = male, 3 = diverse.  *p < .05. **p < .01. ***p < .001 | | | | | | | | | | | |

| **Table S6** *Descriptive Statistics, Correlations, and Scale Reliabilities for Estonia* | | | | | | | | | | | |
| --- | --- | --- | --- | --- | --- | --- | --- | --- | --- | --- | --- |
|  | *M* | *SD* | 1 | 2 | 3 | 4 | 5 | 6 | 7 | 8 | α |
| 1 Gender | 1.48 | 0.50 | 1.00 |  |  |  |  |  |  |  |  |
| 2 Age | 26.87 | 5.44 | .13 | 1.00 |  |  |  |  |  |  |  |
| 3 Competence | 3.47 | 0.92 | .11 | .18 | 1.00 |  |  |  |  |  | .80 |
| 4 Autonomy | 3.53 | 1.05 | .04 | -.12 | .52^**^ | 1.00 |  |  |  |  | .78 |
| 5 Social relatedness | 2.99 | 0.84 | -.05 | .21^*^ | .21^*^ | .29^**^ | 1.00 |  |  |  | .72 |
| 6 Intrinsic motivation | 2.87 | 1.08 | .15 | .23^*^ | .62^**^ | .63^**^ | .32^**^ | 1.00 |  |  | .92 |
| 7 Procrastination | 3.10 | 1.06 | -.01 | -.03 | -.49^**^ | -.42^**^ | -.03 | -.51^**^ | 1.00 |  | .81 |
| 8 Persistence | 3.21 | 0.81 | -.05 | .26^*^ | .40^**^ | .52^**^ | .13 | .42^**^ | -.47^**^ | 1.00 | .74 |
| *Note:* Gender was coded as 1 = female, 2 = male, 3 = diverse.  *p < .05. **p < .01. ***p < .001 | | | | | | | | | | | |

| **Table S7** *Descriptive Statistics, Correlations, and Scale Reliabilities for Finland* | | | | | | | | | | | |
| --- | --- | --- | --- | --- | --- | --- | --- | --- | --- | --- | --- |
|  | *M* | *SD* | 1 | 2 | 3 | 4 | 5 | 6 | 7 | 8 | α |
| 1 Gender | 1.23 | 0.47 | 1.00 |  |  |  |  |  |  |  |  |
| 2 Age | 28.49 | 8.93 | .04 | 1.00 |  |  |  |  |  |  |  |
| 3 Competence | 3.62 | 1.03 | -.09** | .01 | 1.00 |  |  |  |  |  | .90 |
| 4 Autonomy | 3.50 | 1.01 | -.04 | .06^*^ | .46^**^ | 1.00 |  |  |  |  | .76 |
| 5 Social relatedness | 3.40 | 0.90 | -.12^**^ | -.10^**^ | .36^**^ | .20^**^ | 1.00 |  |  |  | .74 |
| 6 Intrinsic motivation | 3.40 | 0.94 | -.01 | .16^**^ | .45^**^ | .39^**^ | .31^**^ | 1.00 |  |  | .86 |
| 7 Procrastination | 3.20 | 1.11 | .06^*^ | -.10^**^ | -.50^**^ | -.24^**^ | -.19^**^ | -.31^**^ | 1.00 |  | .88 |
| 8 Persistence | 2.99 | 0.89 | -.04 | .05^*^ | .51^**^ | .30^**^ | .21^**^ | .33^**^ | -.55^**^ | 1.00 | .83 |
| *Note:* Gender was coded as 1 = female, 2 = male, 3 = diverse.  *p < .05. **p < .01. ***p < .001 | | | | | | | | | | | |

| **Table S8** *Descriptive Statistics, Correlations, and Scale Reliabilities for Germany* | | | | | | | | | | | |
| --- | --- | --- | --- | --- | --- | --- | --- | --- | --- | --- | --- |
|  | *M* | *SD* | 1 | 2 | 3 | 4 | 5 | 6 | 7 | 8 | α |
| 1 Gender | 1.35 | 0.50 | 1.00 |  |  |  |  |  |  |  |  |
| 2 Age | 23.54 | 4.48 | .02 | 1.00 |  |  |  |  |  |  |  |
| 3 Competence | 3.23 | 0.91 | .04 | .04 | 1.00 |  |  |  |  |  | .77 |
| 4 Autonomy | 3.03 | 1.04 | .04 | .03 | .59^**^ | 1.00 |  |  |  |  | .72 |
| 5 Social relatedness | 2.91 | 0.85 | -.08^*^ | -.08^*^ | .22^**^ | .15^**^ | 1.00 |  |  |  | .64 |
| 6 Intrinsic motivation | 2.65 | 1.07 | .03 | .04 | .58^**^ | .54^**^ | .21^**^ | 1.00 |  |  | .90 |
| 7 Procrastination | 3.09 | 1.21 | .11^**^ | .05 | -.40^**^ | -.34^**^ | -.12^**^ | -.39^**^ | 1.00 |  | .91 |
| 8 Persistence | 3.29 | 0.89 | .05 | -.05 | .41^**^ | .34^**^ | .13^**^ | .37^**^ | -.56^**^ | 1.00 | .78 |
| *Note:* Gender was coded as 1 = female, 2 = male, 3 = diverse.  *p < .05. **p < .01. ***p < .001 | | | | | | | | | | | |

| **Table S9** *Descriptive Statistics, Correlations, and Scale Reliabilities for Iceland* | | | | | | | | | | | |
| --- | --- | --- | --- | --- | --- | --- | --- | --- | --- | --- | --- |
|  | *M* | *SD* | 1 | 2 | 3 | 4 | 5 | 6 | 7 | 8 | α |
| 1 Gender | 1.18 | 0.39 | 1.00 |  |  |  |  |  |  |  |  |
| 2 Age | 32.06 | 9.64 | .07 | 1.00 |  |  |  |  |  |  |  |
| 3 Competence | 3.53 | 0.91 | .02 | .11^*^ | 1.00 |  |  |  |  |  | .85 |
| 4 Autonomy | 3.54 | 0.92 | -.01 | .08 | .64^**^ | 1.00 |  |  |  |  | .73 |
| 5 Social relatedness | 3.37 | 0.89 | -.07 | -.07 | .25^*^ | .25^**^ | 1.00 |  |  |  | .69 |
| 6 Intrinsic motivation | 3.23 | 1.11 | -.00 | .14^**^ | .56^**^ | .49^**^ | .29^**^ | 1.00 |  |  | .92 |
| 7 Procrastination | 3.20 | 1.13 | -.09 | -.15^**^ | -.41^**^ | -.28^**^ | -.07 | -.37^**^ | 1.00 |  | .88 |
| 8 Persistence | 3.31 | 0.90 | .03 | .05 | .37^**^ | .30^**^ | .20^**^ | .34^**^ | -.40^**^ | 1.00 | .78 |
| *Note:* Gender was coded as 1 = female, 2 = male, 3 = diverse.  *p < .05. **p < .01. ***p < .001 | | | | | | | | | | | |

| **Table S10** *Descriptive Statistics, Correlations, and Scale Reliabilities for Japan* | | | | | | | | | | | |
| --- | --- | --- | --- | --- | --- | --- | --- | --- | --- | --- | --- |
|  | *M* | *SD* | 1 | 2 | 3 | 4 | 5 | 6 | 7 | 8 | α |
| 1 Gender | 1.34 | 0.49 | 1.00 |  |  |  |  |  |  |  |  |
| 2 Age | 19.69 | 2.54 | .13^**^ | 1.00 |  |  |  |  |  |  |  |
| 3 Competence | 2.68 | 0.90 | .02 | .02 | 1.00 |  |  |  |  |  | .86 |
| 4 Autonomy | 2.97 | 0.94 | .04 | .05 | .71^**^ | 1.00 |  |  |  |  | .62 |
| 5 Social relatedness | 2.68 | 0.90 | -.12^**^ | -.04 | .49^**^ | .38^**^ | 1.00 |  |  |  | .71 |
| 6 Intrinsic motivation | 2.49 | 0.86 | -.08 | .06 | .55^**^ | .57^**^ | .37^**^ | 1.00 |  |  | .85 |
| 7 Procrastination | 3.10 | 1.12 | .14^**^ | .10^*^ | -.29^**^ | -.26^**^ | -.02 | -.26^**^ | 1.00 |  | .85 |
| 8 Persistence | 3.12 | 0.92 | -.02 | -.15^**^ | .30^**^ | .28^**^ | .21^**^ | .26^**^ | -.21^**^ | 1.00 | .76 |
| *Note:* Gender was coded as 1 = female, 2 = male, 3 = diverse.  *p < .05. **p < .01. ***p < .001 | | | | | | | | | | | |

| **Table S11** *Descriptive Statistics, Correlations, and Scale Reliabilities for Kosovo* | | | | | | | | | | | |
| --- | --- | --- | --- | --- | --- | --- | --- | --- | --- | --- | --- |
|  | *M* | *SD* | 1 | 2 | 3 | 4 | 5 | 6 | 7 | 8 | α |
| 1 Gender | 1.28 | 0.46 | 1.00 |  |  |  |  |  |  |  |  |
| 2 Age | 20.38 | 2.21 | .10^**^ | 1.00 |  |  |  |  |  |  |  |
| 3 Competence | 3.94 | 0.83 | .07^*^ | .02 | 1.00 |  |  |  |  |  | .85 |
| 4 Autonomy | 4.07 | 0.82 | .07^*^ | -.00 | .70^**^ | 1.00 |  |  |  |  | .79 |
| 5 Social relatedness | 3.96 | 0.75 | -.04 | -.05 | .45^**^ | .41^**^ | 1.00 |  |  |  | .75 |
| 6 Intrinsic motivation | 3.93 | 0.91 | .02 | -.01 | .58^**^ | .48^**^ | .38^**^ | 1.00 |  |  | .88 |
| 7 Procrastination | 3.00 | 1.10 | .03 | .02 | -.29^**^ | -.22^**^ | -.14^**^ | -.27^**^ | 1.00 |  | .88 |
| 8 Persistence | 3.99 | 0.84 | -.02 | -.09^**^ | .48^**^ | .40^**^ | .34^**^ | .43^**^ | -.26^**^ | 1.00 | .86 |
| *Note:* Gender was coded as 1 = female, 2 = male, 3 = diverse.  *p < .05. **p < .01. ***p < .001 | | | | | | | | | | | |

| **Table S12** *Descriptive Statistics, Correlations, and Scale Reliabilities for Lithuania* | | | | | | | | | | | |
| --- | --- | --- | --- | --- | --- | --- | --- | --- | --- | --- | --- |
|  | *M* | *SD* | 1 | 2 | 3 | 4 | 5 | 6 | 7 | 8 | α |
| 1 Gender | 1.09 | 0.29 | 1.00 |  |  |  |  |  |  |  |  |
| 2 Age | 25.55 | 7.71 | -.07 | 1.00 |  |  |  |  |  |  |  |
| 3 Competence | 3.29 | 0.96 | .02 | .11 | 1.00 |  |  |  |  |  | .82 |
| 4 Autonomy | 3.59 | 0.93 | .04 | -.12 | .52^**^ | 1.00 |  |  |  |  | .78 |
| 5 Social relatedness | 3.54 | 0.87 | -.01 | .14^*^ | .35^**^ | .07 | 1.00 |  |  |  | .70 |
| 6 Intrinsic motivation | 2.95 | 0.98 | .03 | .11 | .52^**^ | .41^**^ | .32^**^ | 1.00 |  |  | .84 |
| 7 Procrastination | 3.13 | 1.16 | .16^**^ | -.14^*^ | -.50^**^ | -.26^**^ | -.19^**^ | -.25^**^ | 1.00 |  | .90 |
| 8 Persistence | 3.35 | 0.91 | -.09 | .06 | .46^**^ | .26^**^ | .14^*^ | .29^**^ | -.48^**^ | 1.00 | .81 |
| *Note:* Gender was coded as 1 = female, 2 = male, 3 = diverse.  *p < .05. **p < .01. ***p < .001 | | | | | | | | | | | |

| **Table S13** *Descriptive Statistics, Correlations, and Scale Reliabilities for Malta* | | | | | | | | | | | |
| --- | --- | --- | --- | --- | --- | --- | --- | --- | --- | --- | --- |
|  | *M* | *SD* | 1 | 2 | 3 | 4 | 5 | 6 | 7 | 8 | α |
| 1 Gender | 1.31 | 0.46 | 1.00 |  |  |  |  |  |  |  |  |
| 2 Age | 23.63 | 7.71 | .01 | 1.00 |  |  |  |  |  |  |  |
| 3 Competence | 2.99 | 0.97 | .04 | .16^*^ | 1.00 |  |  |  |  |  | .86 |
| 4 Autonomy | 3.25 | 0.95 | .02 | .19^**^ | .71^**^ | 1.00 |  |  |  |  | .82 |
| 5 Social relatedness | 3.18 | 0.83 | -.13 | .19^**^ | .33^**^ | .40^**^ | 1.00 |  |  |  | .70 |
| 6 Intrinsic motivation | 2.32 | 0.96 | .03 | .28^**^ | .65^**^ | .59^**^ | .27^**^ | 1.00 |  |  | .89 |
| 7 Procrastination | 2.97 | 1.13 | -.01 | -.11 | -.50^**^ | -.43^**^ | -.21^**^ | -.41^**^ | 1.00 |  | .90 |
| 8 Persistence | 2.82 | 0.94 | .06 | .10 | .38^**^ | .43^**^ | .18^**^ | .43^**^ | -.52^**^ | 1.00 | .82 |
| *Note:* Gender was coded as 1 = female, 2 = male, 3 = diverse.  *p < .05. **p < .01. ***p < .001 | | | | | | | | | | | |

| **Table S14** *Descriptive Statistics, Correlations, and Scale Reliabilities for North Macedonia* | | | | | | | | | | | |
| --- | --- | --- | --- | --- | --- | --- | --- | --- | --- | --- | --- |
|  | *M* | *SD* | 1 | 2 | 3 | 4 | 5 | 6 | 7 | 8 | α |
| 1 Gender | 1.16 | 0.37 | 1.00 |  |  |  |  |  |  |  |  |
| 2 Age | 21.79 | 3.73 | .15^*^ | 1.00 |  |  |  |  |  |  |  |
| 3 Competence | 3.80 | 0.84 | .12 | .11 | 1.00 |  |  |  |  |  | .74 |
| 4 Autonomy | 3.59 | 0.94 | .11 | .06 | .67^**^ | 1.00 |  |  |  |  | .67 |
| 5 Social relatedness | 3.60 | 0.86 | -.02 | .10 | .38^**^ | .36^**^ | 1.00 |  |  |  | .71 |
| 6 Intrinsic motivation | 3.05 | 0.97 | -.10 | .13^*^ | .51^**^ | .47^**^ | .36^**^ | 1.00 |  |  | .83 |
| 7 Procrastination | 3.01 | 1.08 | .16^*^ | .04 | -.49^**^ | -.34^**^ | -.29^**^ | -.49^**^ | 1.00 |  | .85 |
| 8 Persistence | 3.55 | 0.93 | .05 | .04 | .50^**^ | .41^**^ | .35^**^ | .41^**^ | -.50^**^ | 1.00 | .82 |
| *Note:* Gender was coded as 1 = female, 2 = male, 3 = diverse.  *p < .05. **p < .01. ***p < .001 | | | | | | | | | | | |

| **Table S15** *Descriptive Statistics, Correlations, and Scale Reliabilities for Poland* | | | | | | | | | | | |
| --- | --- | --- | --- | --- | --- | --- | --- | --- | --- | --- | --- |
|  | *M* | *SD* | 1 | 2 | 3 | 4 | 5 | 6 | 7 | 8 | α |
| 1 Gender | 1.16 | 0.38 | 1.00 |  |  |  |  |  |  |  |  |
| 2 Age | 22.61 | 4.16 | .14^**^ | 1.00 |  |  |  |  |  |  |  |
| 3 Competence | 3.35 | 0.84 | .01 | .10^*^ | 1.00 |  |  |  |  |  | .82 |
| 4 Autonomy | 3.14 | 0.92 | .00 | .03 | .56^**^ | 1.00 |  |  |  |  | .67 |
| 5 Social relatedness | 3.71 | 0.80 | -.17^**^ | -.04 | .33^**^ | .30^**^ | 1.00 |  |  |  | .69 |
| 6 Intrinsic motivation | 2.79 | 0.97 | -.02 | .09^*^ | .62^**^ | .56^**^ | .30^**^ | 1.00 |  |  | .88 |
| 7 Procrastination | 3.13 | 1.08 | .10^*^ | -.04 | -.39^**^ | -.27^**^ | -.15^**^ | -.28^**^ | 1.00 |  | .86 |
| 8 Persistence | 3.18 | 0.83 | -.03 | .05 | .38^**^ | .25^**^ | .17^**^ | .26^**^ | -.48^**^ | 1.00 | .79 |
| *Note:* Gender was coded as 1 = female, 2 = male, 3 = diverse.  *p < .05. **p < .01. ***p < .001 | | | | | | | | | | | |

| **Table S16** *Descriptive Statistics, Correlations, and Scale Reliabilities for Romania* | | | | | | | | | | | |
| --- | --- | --- | --- | --- | --- | --- | --- | --- | --- | --- | --- |
|  | *M* | *SD* | 1 | 2 | 3 | 4 | 5 | 6 | 7 | 8 | α |
| 1 Gender | 1.25 | 0.43 | 1.00 |  |  |  |  |  |  |  |  |
| 2 Age | 20.86 | 2.70 | .02 | 1.00 |  |  |  |  |  |  |  |
| 3 Competence | 3.39 | 0.90 | .02 | .10 | 1.00 |  |  |  |  |  | .87 |
| 4 Autonomy | 3.48 | 0.94 | .09 | .12^*^ | .73^**^ | 1.00 |  |  |  |  | .83 |
| 5 Social relatedness | 3.54 | 0.84 | -.04 | .03 | .54^**^ | .47^**^ | 1.00 |  |  |  | .75 |
| 6 Intrinsic motivation | 3.07 | 0.88 | -.01 | .07 | .62^**^ | .56^**^ | .33^**^ | 1.00 |  |  | .88 |
| 7 Procrastination | 3.39 | 0.96 | -.03 | -.12^*^ | -.33^**^ | -.31^**^ | -.19^**^ | -.30^**^ | 1.00 |  | .83 |
| 8 Persistence | 3.53 | 0.87 | .07 | .06 | .42^**^ | .44^**^ | .16^**^ | .39^**^ | -.45^**^ | 1.00 | .84 |
| *Note:* Gender was coded as 1 = female, 2 = male, 3 = diverse.  *p < .05. **p < .01. ***p < .001 | | | | | | | | | | | |

| **Table S17** *Descriptive Statistics, Correlations, and Scale Reliabilities for Sweden* | | | | | | | | | | | |
| --- | --- | --- | --- | --- | --- | --- | --- | --- | --- | --- | --- |
|  | *M* | *SD* | 1 | 2 | 3 | 4 | 5 | 6 | 7 |  | α |
| 1 Gender | 1.31 | 0.49 | 1.00 |  |  |  |  |  |  |  |  |
| 2 Competence | 3.58 | 0.91 | -.05^*^ | 1.00 |  |  |  |  |  |  | .84 |
| 3 Autonomy | 3.52 | 0.91 | -.07^**^ | .66^**^ | 1.00 |  |  |  |  |  | .79 |
| 4 Social relatedness | 3.22 | 0.89 | -.06^*^ | .35^**^ | .33^**^ | 1.00 |  |  |  |  | .70 |
| 5 Intrinsic motivation | 3.18 | 1.10 | -.07^**^ | .56^**^ | .56^**^ | .40^**^ | 1.00 |  |  |  | .94 |
| 6 Procrastination | 2.84 | 1.15 | .09^**^ | -.42^**^ | -.44^**^ | -.23^**^ | -.35^**^ | 1.00 |  |  | .90 |
| 7 Persistence | 3.43 | 0.87 | -.03 | .46^**^ | .47^**^ | .27^**^ | .38^**^ | -.55^**^ | 1.00 |  | .80 |
| *Note:* Gender was coded as 1 = female, 2 = male, 3 = diverse. In Sweden, age was only measured as a categorical variable. Analyses were conducted for all countries with that same categorized variable, but to include its mean and correlation for Sweden is nonsensical.  *p < .05. **p < .01. ***p < .001 | | | | | | | | | | | |

| **Table S18** *Descriptive Statistics, Correlations, and Scale Reliabilities for USA* | | | | | | | | | | | |
| --- | --- | --- | --- | --- | --- | --- | --- | --- | --- | --- | --- |
|  | *M* | *SD* | 1 | 2 | 3 | 4 | 5 | 6 | 7 | 8 | α |
| 1 Gender | 1.47 | 0.53 | 1.00 |  |  |  |  |  |  |  |  |
| 2 Age | 20.23 | 2.17 | .08 | 1.00 |  |  |  |  |  |  |  |
| 3 Competence | 3.06 | 1.36 | -.09^*^ | -.08 | 1.00 |  |  |  |  |  | .87 |
| 4 Autonomy | 4.37 | 1.05 | -.08 | -.01 | .07 | 1.00 |  |  |  |  | .84 |
| 5 Social relatedness | 3.55 | 1.47 | -.09^*^ | -.04 | .46^**^ | .00 | 1.00 |  |  |  | .88 |
| 6 Intrinsic motivation | 3.01 | 1.37 | -.00 | -.07 | -.07 | -.05 | .05 | 1.00 |  |  | .85 |
| 7 Procrastination | 3.40 | 1.10 | .11^*^ | .02 | -.04 | -.01 | -.00 | -.03 | 1.00 |  | .74 |
| 8 Persistence | 3.45 | 1.08 | .06 | -.05 | -.08 | -.02 | -.01 | -.02 | .02 | 1.00 | .79 |
| *Note:* Gender was coded as 1 = female, 2 = male, 3 = diverse.  *p < .05. **p < .01. ***p < .001 | | | | | | | | | | | |

| **Table S19**  *Measurement Invariance Testing for the Confirmatory Factor Analytic Measurement Models – fit indices per variable* | | | | | | |
| --- | --- | --- | --- | --- | --- | --- |
| Model description | | *χ2* | *df* | CFI | RMSEA | BIC |
| Autonomy + Competence | | | | | | |
|  | Configural invariance | 336.973* | 68 | - | - | 202,847.525 |
|  | Metric invariance | 614.514* | 116 | 0.978 | 0.069 | 202,691.416 |
|  | Scalar invariance | 2,026.173* | 164 | 0.919 | 0.112 | 203,800.454 |
| Autonomy + Social relatedness | | | | | | |
|  | Configural invariance | 374.481* | 68 | - | - | 216,716.063 |
|  | Metric invariance | 574.101* | 116 | 0.973 | 0.066 | 216,489.205 |
|  | Scalar invariance | 1,926.512* | 164 | 0.895 | 0.109 | 217,535.550 |
| Competence | | | | | | |
|  | Configural invariance | 0.003* | 0 | - | - | 123,277.603 |
|  | Metric invariance | 182.376* | 32 | 0.986 | 0.072 | 123,171.838 |
|  | Scalar invariance | 1,249.324* | 64 | 0.893 | 0.143 | 123,987.240 |
| Social relatedness | | | | | | |
|  | Configural invariance | 0.000* | 0 | - | - | 130,075.500 |
|  | Metric invariance | 172.920* | 32 | 0.984 | 0.070 | 129,975.658 |
|  | Scalar invariance | 1,133.621* | 64 | 0.881 | 0.136 | 130,750.220 |
| Intrinsic motivation | | | | | | |
|  | Configural invariance | 0.003* | 0 | - | - | 115,132.199 |
|  | Metric invariance | 117.846* | 32 | 0.995 | 0.054 | 114,938.872 |
|  | Scalar invariance | 1,321.342* | 64 | 0.925 | 0.147 | 115,828.483 |
| Passive procrastination | | | | | | |
|  | Configural invariance | 0.008* | 0 | - | - | 126,122.835 |
|  | Metric invariance | 195.257* | 32 | 0.988 | 0.076 | 126,014.894 |
|  | Scalar invariance | 1,114.115* | 64 | 0.924 | 0.136 | 126,650.932 |
| Persistence | | | | | | |
|  | Configural invariance | 0.001* | 0 | - | - | 117,911.682 |
|  | Metric invariance | 722.572* | 32 | 0.993 | 0.081 | 117,849.375 |
|  | Scalar invariance | 1,709.991* | 64 | 0.948 | 0.190 | 118,582.745 |
| *Note*. BIC = Bayesian Information Criterion; χ2 = chi square test of model fit; CFI = comparative fit index; RMSEA = root mean square error of approximation.  *p ≤ .05 | | | | | | |

| **Table S20**  *Effects of gender and age on intrinsic motivation, procrastination, and persistence in the autonomy model* | | | | | | | | | | | | | |  |
| --- | --- | --- | --- | --- | --- | --- | --- | --- | --- | --- | --- | --- | --- | --- |
|  |  | Intrinsic motivation | | | | Procrastination | | | | Persistence | | | |  |
|  |  | | Est. | *SE* | *p* | | Est. | *SE* | *p* | | Est. | *SE* | *p* | |
| Albania | Gender | | -.11 | .09 | .22 | | -.04 | .13 | .74 | | -.07 | .09 | .43 | |
|  | Age | | .10 | .07 | .12 | | .12 | .11 | .28 | | .07 | .06 | .24 | |
| Austria | Gender | | -.01 | .03 | .77 | | .03 | .03 | .34 | | .04 | .02 | .07 | |
|  | Age | | .05 | .02 | .00 | | -.01 | .02 | .75 | | .02 | .01 | .13 | |
| China | Gender | | .02 | .08 | .80 | | .09 | .11 | .42 | | .14 | .06 | .03 | |
|  | Age | | .04 | .06 | .55 | | .08 | .09 | .34 | | .09 | .05 | .09 | |
| Croatia | Gender | | -.10 | .12 | .40 | | .15 | .13 | .23 | | -.01 | .08 | .88 | |
|  | Age | | .09 | .06 | .16 | | -.07 | .08 | .37 | | .02 | .04 | .63 | |
| Estonia | Gender | | .23 | .16 | .17 | | .07 | .18 | .69 | | -.07 | .12 | .55 | |
|  | Age | | .02 | .12 | .89 | | .16 | .14 | .23 | | .13 | .08 | .12 | |
| Finland | Gender | | -.00 | .05 | .99 | | .09 | .06 | .14 | | -.05 | .04 | .19 | |
|  | Age | | .11 | .02 | .00 | | -.06 | .03 | .03 | | .01 | .02 | .66 | |
| Germany | Gender | | .02 | .07 | .79 | | .30 | .09 | .00 | | -.03 | .05 | .52 | |
|  | Age | | .05 | .06 | .40 | | .18 | .07 | .01 | | -.06 | .04 | .19 | |
| Iceland | Gender | | -.00 | .14 | .99 | | -.20 | .13 | .12 | | .07 | .09 | .47 | |
|  | Age | | .12 | .05 | .02 | | -.10 | .05 | .06 | | .04 | .04 | .24 | |
| Japan | Gender | | -.20 | .06 | .00 | | .33 | .10 | .00 | | -.00 | .06 | .97 | |
|  | Age | | .43 | .13 | .00 | | .21 | .27 | .44 | | -.39 | .10 | .00 | |
| Kosovo | Gender | | -.03 | .06 | .63 | | .12 | .08 | .11 | | -.02 | .05 | .71 | |
|  | Age | | .05 | .09 | .55 | | .02 | .13 | .87 | | -.26 | .08 | .00 | |
| Lithuania | Gender | | .07 | .18 | .69 | | .69 | .19 | .00 | | -.26 | .14 | .05 | |
|  | Age | | .19 | .07 | .01 | | -.12 | .07 | .09 | | .03 | .06 | .57 | |
| Malta | Gender | | .01 | .13 | .93 | | .01 | .16 | .95 | | .04 | .11 | .74 | |
|  | Age | | .17 | .07 | .01 | | -.03 | .07 | .70 | | .01 | .07 | .92 | |
| North Macedonia | Gender | | -.46 | .19 | .02 | | .44 | .21 | .03 | | .01 | .15 | .94 | |
|  | Age | | .26 | .17 | .14 | | .08 | .14 | .60 | | .04 | .12 | .73 | |
| Poland | Gender | | -.09 | .08 | .26 | | .35 | .13 | .01 | | -.03 | .07 | .66 | |
|  | Age | | .20 | .06 | .00 | | -.10 | .08 | .22 | | .06 | .06 | .35 | |
| Romania | Gender | | -.16 | .09 | .09 | | -.02 | .12 | .87 | | .11 | .08 | .14 | |
|  | Age | | .10 | .18 | .57 | | -.28 | .17 | .10 | | .06 | .13 | .63 | |
| Sweden | Gender | | -.09 | .05 | .06 | | .15 | .05 | .00 | | .03 | .03 | .41 | |
|  | Age | | .17 | .02 | .00 | | -.04 | .02 | .07 | | .03 | .02 | .05 | |
| USA | Gender | | .07 | .14 | .61 | | .23 | .10 | .02 | | .15 | .08 | .07 | |
|  | Age | | -.30 | .30 | .33 | | .16 | .32 | .62 | | -.24 | .25 | .33 | |
| *Note.* Gender was coded as 1 = female, 2 = male, 3 = diverse. | | | | | | | | | | | | | | |

| **Table S21**  *Effects of gender and age on intrinsic motivation, procrastination, and persistence in the competence model* | | | | | | | | | | |
| --- | --- | --- | --- | --- | --- | --- | --- | --- | --- | --- |
|  |  | Intrinsic motivation | | | Procrastination | | | Persistence | | |
|  |  | Est. | *SE* | *p* | Est. | *SE* | *p* | Est. | *SE* | *p* |
| Albania | Gender | -.12 | .08 | .14 | -.06 | .13 | .64 | -.07 | .09 | .46 |
|  | Age | -.01 | .05 | .80 | .21 | .11 | .07 | .01 | .06 | .87 |
| Austria | Gender | -.01 | .02 | .67 | .04 | .03 | .25 | .03 | .02 | .10 |
|  | Age | .04 | .02 | .02 | .00 | .02 | .97 | .01 | .01 | .23 |
| China | Gender | -.03 | .07 | .73 | .12 | .11 | .29 | .12 | .06 | .07 |
|  | Age | .14 | .05 | .01 | .03 | .09 | .71 | .13 | .05 | .01 |
| Croatia | Gender | -.04 | .11 | .74 | .13 | .13 | .31 | .01 | .08 | .91 |
|  | Age | .08 | .06 | .17 | -.08 | .07 | .29 | .02 | .04 | .58 |
| Estonia | Gender | .19 | .16 | .26 | .07 | .18 | .70 | -.07 | .13 | .58 |
|  | Age | .19 | .11 | .09 | .09 | .14 | .50 | .19 | .10 | .05 |
| Finland | Gender | .08 | .05 | .10 | .03 | .05 | .60 | -.01 | .04 | .81 |
|  | Age | .13 | .02 | .00 | -.08 | .03 | .00 | .02 | .02 | .16 |
| Germany | Gender | .04 | .07 | .54 | .29 | .08 | .00 | -.02 | .05 | .64 |
|  | Age | .04 | .06 | .50 | .18 | .07 | .01 | -.06 | .04 | .19 |
| Iceland | Gender | -.03 | .13 | .80 | -.19 | .13 | .15 | .06 | .09 | .55 |
|  | Age | .10 | .05 | .04 | -.10 | .05 | .06 | .03 | .03 | .35 |
| Japan | Gender | -.18 | .07 | .01 | .35 | .10 | .00 | -.01 | .06 | .89 |
|  | Age | .47 | .17 | .00 | .14 | .26 | .59 | -.35 | .11 | .00 |
| Kosovo | Gender | -.03 | .06 | .64 | .13 | .07 | .07 | -.02 | .04 | .62 |
|  | Age | .03 | .08 | .70 | .02 | .14 | .87 | -.26 | .09 | .00 |
| Lithuania | Gender | .07 | .18 | .69 | .69 | .18 | .00 | -.26 | .14 | .06 |
|  | Age | .04 | .06 | .48 | -.07 | .07 | .28 | -.02 | .06 | .78 |
| Malta | Gender | -.02 | .12 | .88 | .03 | .15 | .85 | .02 | .11 | .85 |
|  | Age | .17 | .06 | .01 | -.04 | .07 | .61 | .01 | .07 | .90 |
| North Macedonia | Gender | -.45 | .18 | .01 | .50 | .20 | .01 | -.02 | .14 | .87 |
|  | Age | .23 | .16 | .15 | .07 | .16 | .66 | .04 | .12 | .73 |
| Poland | Gender | -.07 | .08 | .39 | .34 | .12 | .00 | -.03 | .07 | .65 |
|  | Age | .11 | .07 | .08 | -.07 | .08 | .36 | .03 | .06 | .61 |
| Romania | Gender | -.07 | .09 | .46 | -.04 | .12 | .75 | .14 | .08 | .08 |
|  | Age | .17 | .14 | .22 | -.30 | .16 | .06 | .08 | .11 | .49 |
| Sweden | Gender | -.10 | .04 | .02 | .16 | .05 | .00 | .02 | .03 | .55 |
|  | Age | .17 | .02 | .00 | -.06 | .02 | .02 | .04 | .02 | .02 |
| USA | Gender | .13 | .15 | .36 | .18 | .10 | .08 | .18 | .08 | .03 |
|  | Age | .16 | .46 | .73 | -.06 | .33 | .86 | -.07 | .24 | .78 |
| *Note.* Gender was coded as 1 = female, 2 = male, 3 = diverse. | | | | | | | | | | |

| **Table S22**  *Effects of gender and age on intrinsic motivation, procrastination, and persistence in the social relatedness model* | | | | | | | | | | |
| --- | --- | --- | --- | --- | --- | --- | --- | --- | --- | --- |
|  |  | Intrinsic motivation | | | Procrastination | | | Persistence | | |
|  |  | Est. | *SE* | *p* | Est. | *SE* | *p* | Est. | *SE* | *p* |
| Albania | Gender | -.26 | .14 | .02 | -.02 | .14 | .88 | -.09 | .09 | .34 |
|  | Age | .26 | .08 | .00 | .10 | .12 | .39 | .08 | .06 | .15 |
| Austria | Gender | .05 | .03 | .10 | .02 | .03 | .54 | .05 | .02 | .02 |
|  | Age | .09 | .02 | .00 | -.01 | .02 | .66 | .02 | .01 | .09 |
| China | Gender | .06 | .08 | .45 | .08 | .11 | .46 | .14 | .07 | .03 |
|  | Age | .17 | .06 | .00 | .06 | .09 | .52 | .11 | .05 | .04 |
| Croatia | Gender | .00 | .11 | .98 | .12 | .13 | .35 | .02 | .08 | .84 |
|  | Age | .05 | .06 | .40 | -.05 | .07 | .52 | .01 | .05 | .84 |
| Estonia | Gender | .29 | .20 | .14 | .09 | .19 | .64 | -.07 | .13 | .56 |
|  | Age | .27 | .13 | .04 | .10 | .14 | .47 | .18 | .09 | .05 |
| Finland | Gender | .01 | .05 | .91 | .09 | .06 | .15 | -.05 | .04 | .20 |
|  | Age | .18 | .02 | .00 | -.06 | .03 | .03 | .01 | .02 | .48 |
| Germany | Gender | .10 | .08 | .24 | .29 | .09 | .00 | -.02 | .05 | .71 |
|  | Age | .10 | .07 | .16 | .18 | .07 | .01 | -.05 | .04 | .25 |
| Iceland | Gender | .01 | .15 | .93 | -.21 | .14 | .13 | .07 | .10 | .45 |
|  | Age | .18 | .05 | .00 | -.10 | .05 | .06 | .04 | .03 | .26 |
| Japan | Gender | -.11 | .07 | .13 | .29 | .10 | .00 | .04 | .06 | .51 |
|  | Age | .41 | .27 | .13 | .26 | .28 | .35 | -.43 | .10 | .00 |
| Kosovo | Gender | .05 | .06 | .39 | .10 | .08 | .19 | .00 | .05 | .92 |
|  | Age | .05 | .09 | .58 | .03 | .14 | .83 | -.26 | .08 | .00 |
| Lithuania | Gender | .13 | .17 | .46 | .68 | .19 | .00 | -.25 | .15 | .08 |
|  | Age | .06 | .07 | .41 | -.07 | .08 | .34 | -.01 | .06 | .82 |
| Malta | Gender | .09 | .15 | .56 | -.01 | .16 | .97 | .05 | .11 | .65 |
|  | Age | .22 | .09 | .01 | -.03 | .08 | .74 | .00 | .07 | .97 |
| North Macedonia | Gender | -.34 | .20 | .09 | .36 | .20 | .08 | .08 | .15 | .57 |
|  | Age | .29 | .16 | .06 | .10 | .16 | .53 | .02 | .12 | .86 |
| Poland | Gender | .01 | .09 | .96 | .32 | .13 | .01 | .00 | .07 | 1.00 |
|  | Age | .22 | .08 | .00 | -.08 | .09 | .32 | .04 | .06 | .51 |
| Romania | Gender | -.02 | .10 | .82 | -.07 | .12 | .56 | .15 | .08 | .05 |
|  | Age | .31 | .21 | .13 | -.32 | .17 | .05 | .09 | .12 | .44 |
| Sweden | Gender | -.13 | .05 | .01 | .15 | .06 | .01 | .03 | .03 | .40 |
|  | Age | .23 | .02 | .00 | -.04 | .03 | .12 | .03 | .02 | .13 |
| USA | Gender | .05 | .13 | .72 | .25 | .10 | .02 | .13 | .08 | .11 |
|  | Age | -.21 | .33 | .52 | .11 | .34 | .76 | -.19 | .26 | .45 |
| *Note.* Gender was coded as 1 = female, 2 = male, 3 = diverse. | | | | | | | | | | |

| **Table S23**  *Unstandardized path coefficients, standard errors, p-values, and two-sided 5 % confidence intervals for the multigroup mediation models predicting procrastination* | | | | | | | | | | | | | |  |
| --- | --- | --- | --- | --- | --- | --- | --- | --- | --- | --- | --- | --- | --- | --- |
| Country |  | Autonomy | | | | Competence | | | | Social Relatedness | | | |  |
|  |  | Est. | *S.E.* | *p* | CI | Est. | *S.E.* | *p* | CI | Est. | *S.E.* | *p* | CI |  |
| Albania | Direct | -0.281 | 0.160 | .079 | (-0.593\|0.042) | -0.120 | 0.213 | .573 | (-0.575\|0.275) | 0.095 | 0.071 | .185 | (-0.050\|0.230) |  |
|  | Indirect | -0.344 | 0.140 | .014 | (-0.632\|-0.078) | -0.460 | 0.205 | .025 | (-0.847\|-0.032) | -0.428 | 0.060 | <.001 | (-0.555\|-0.319) |  |
| Austria | Direct | -0.091 | 0.032 | .004 | (-0.154\|-0.029) | -0.476 | 0.034 | <.001 | (-0.539\|-0.410) | -0.059 | 0.016 | <.001 | (-0.090\|-0.028) |  |
|  | Indirect | -0.310 | 0.022 | <.001 | (-0.352\|-0.268) | -0.107 | 0.023 | <.001 | (-0.153\|-0.062) | -0.099 | 0.007 | <.001 | (-0.113\|-0.085) |  |
| China | Direct | -0.235 | 0.123 | .056 | (-0.473\|0.008) | -0.145 | 0.162 | .370 | (-0.461\|0.167) | -0.032 | 0.082 | .699 | (-0.195\|0.125) |  |
|  | Indirect | 0.036 | 0.066 | .582 | (-0.101\|0.162) | 0.012 | 0.104 | .911 | (-0.190\|0.216) | -0.030 | 0.051 | .566 | (-0.124\|0.076) |  |
| Croatia | Direct | -0.136 | 0.109 | .212 | (-0.338\|0.095) | -0.204 | 0.106 | .055 | (-0.421\|0.000) | 0.050 | 0.101 | .619 | (-0.144\|0.251) |  |
|  | Indirect | -0.286 | 0.069 | <.001 | (-0.445\|-0.169) | -0.254 | 0.069 | <.001 | (-0.392\|-0.124) | -0.287 | 0.058 | <.001 | (-0.417\|-0.186) |  |
| Estonia | Direct | -0.286 | 0.390 | .463 | (-1.060\|0.115) | -0.400 | 0.234 | .086 | (-0.868\|0.027) | 0.359 | 0.136 | .008 | (0.092\|0.619) |  |
|  | Indirect | -0.364 | 0.323 | .259 | (-0.802\|0.023) | -0.335 | 0.166 | .043 | (-0.698\|-0.046) | -0.268 | 0.106 | .011 | (-0.508\|-0.076) |  |
| Finland | Direct | -0.187 | 0.047 | <.001 | (-0.279\|-0.101) | -0.511 | 0.035 | <.001 | (-0.579\|-0.443) | -0.099 | 0.030 | .001 | (-0.157\|-0.040) |  |
|  | Indirect | -0.165 | 0.022 | <.001 | (-0.212\|-0.124) | -0.050 | 0.019 | .008 | (-0.086\|-0.012) | -0.105 | 0.013 | <.001 | (-0.134\|-0.081) |  |
| Germany | Direct | -0.367 | 0.092 | <.001 | (-0.545\|-0.182) | -0.579 | 0.097 | <.001 | (-0.775\|-0.394) | -0.021 | 0.053 | .696 | (-0.123\|0.084) |  |
|  | Indirect | -0.215 | 0.055 | <.001 | (-0.331\|-0.112) | -0.118 | 0.068 | .083 | (-0.249\|0.015) | -0.106 | 0.026 | <.001 | (-0.160\|-0.059) |  |
| Iceland | Direct | -0.245 | 0.143 | .088 | (-0.530\|0.030) | -0.472 | 0.109 | <.001 | (-0.674\|-0.256) | 0.009 | 0.061 | .886 | (-0.112\|0.125) |  |
|  | Indirect | -0.218 | 0.078 | .005 | (-0.381\|-0.070) | -0.106 | 0.069 | .126 | (-0.247\|0.029) | -0.099 | 0.026 | <.001 | (-0.161\|-0.057) |  |
| Japan | Direct | -0.449 | 0.227 | .048 | (-0.969\|-0.088) | -0.280 | 0.082 | .001 | (-0.433\|-0.112) | 0.108 | 0.056 | .055 | (-0.001\|0.217) |  |
|  | Indirect | -0.056 | 0.163 | .730 | (-0.297\|0.309) | -0.105 | 0.049 | .033 | (-0.207\|-0.015) | -0.140 | 0.029 | <.001 | (-0.205\|-0.093) |  |
| Kosovo | Direct | -0.173 | 0.076 | .022 | (-0.328\|-0.028) | -0.283 | 0.074 | <.001 | (-0.426\|-0.139) | -0.018 | 0.051 | .719 | (-0.115\|0.084) |  |
|  | Indirect | -0.214 | 0.042 | <.001 | (-0.300\|-0.133) | -0.151 | 0.049 | .002 | (-0.249\|-0.056) | -0.160 | 0.025 | <.001 | (-0.211\|-0.114) |  |
| Lithuania | Direct | -0.354 | 0.135 | .009 | (-0.618\|-0.095) | -0.746 | 0.119 | <.001 | (-0.989\|-0.518) | -0.107 | 0.079 | .177 | (-0.272\|0.044) |  |
|  | Indirect | -0.110 | 0.065 | .087 | (-0.242\|0.013) | 0.071 | 0.077 | .356 | (-0.059\|0.249) | -0.084 | 0.031 | .006 | (-0.154\|-0.035) |  |
| Malta | Direct | -0.484 | 0.188 | .010 | (-0.871\|-0.136) | -0.584 | 0.137 | <.001 | (-0.819\|-0.288) | -0.035 | 0.099 | .721 | (-0.234\|0.149) |  |
|  | Indirect | -0.170 | 0.103 | .100 | (-0.385\|0.027) | -0.058 | 0.091 | .521 | (-0.256\|0.109) | -0.119 | 0.046 | .010 | (-0.230\|-0.045) |  |
| North Macedonia | Direct | -0.259 | 0.184 | .158 | (-0.631\|0.091) | -0.795 | 0.174 | <.001 | (-1.190\|-0.510) | -1.106 | 0.080 | .184 | (-0.256\|0.057) |  |
|  | Indirect | -0.373 | 0.123 | .002 | (-0.677\|-0.184) | -0.172 | 0.116 | .138 | (-0.376\|0.070) | -0.198 | 0.047 | <.001 | (-0.310\|-0.119) |  |
| Poland | Direct | -0.334 | 0.133 | .012 | (-0.597\|-0.075) | -0.672 | 0.101 | <.001 | (-0.865\|-0.478) | -0.041 | 0.060 | .496 | (-0.159\|0.078) |  |
|  | Indirect | -0.150 | 0.084 | .074 | (-0.316\|0.016) | 0.050 | 0.066 | .448 | (-0.076\|0.182) | -0.110 | 0.024 | <.001 | (-0.166\|-0.069) |  |
| Romania | Direct | -0.245 | 0.115 | .033 | (-0.472\|-0.017) | -0.290 | 0.104 | .005 | (-0.493\|-0.082) | -0.156 | 0.071 | .027 | (-0.287\|-0.010) |  |
|  | Indirect | -0.127 | 0.066 | .054 | (-0.252\|0.006) | -0.092 | 0.069 | .182 | (-0.232\|0.039) | -0.089 | 0.029 | .002 | (-0.157\|-0.042) |  |
| Sweden | Direct | -0.653 | 0.057 | <.001 | (-0.769\|-0.545) | -0.507 | 0.049 | <.001 | (-0.603\|-0.412) | -0.112 | 0.030 | <.001 | (-0.170\|-0.051) |  |
|  | Indirect | -0.063 | 0.034 | .059 | (-0.129\|0.002) | -0.091 | 0.030 | .002 | (-0.149\|-0.034) | -0.135 | 0.015 | <.001 | (-0.166\|-0.107) |  |
| USA | Direct | -0.023 | 0.065 | .723 | (-0.146\|0.108) | -0.016 | 0.042 | .697 | (-0.097\|0.068) | -0.008 | 0.031 | .796 | (-0.067\|0.051) |  |
|  | Indirect | 0.001 | 0.006 | .812 | (-0.006\|0.020) | 0.001 | 0.005 | .782 | (-0.006\|0.014) | 0.000 | 0.002 | .884 | (-0.008\|0.002) |  |
| *Note.* CI = two-sided 5% confidence intervals | | | | | | | | | | | | | | |

| **Table S24**  *Unstandardized path coefficients, standard errors, p-values, and two-sided 5 % confidence intervals for the multigroup mediation models predicting persistence* | | | | | | | | | | | | | | |
| --- | --- | --- | --- | --- | --- | --- | --- | --- | --- | --- | --- | --- | --- | --- |
| Country |  | Autonomy | | | | | Competence | | | | Social Relatedness | | | |
|  |  | Est. | *S.E.* | *p* | CI | | Est. | *S.E.* | *p* | CI | Est. | *S.E.* | *p* | CI |
| Albania | Direct | 0.270 | 0.111 | .015 | (0.060\|0.489) | | 0.208 | 0.168 | .216 | (-0.126\|0.539) | 0.096 | 0.052 | .067 | (-0.003\|0.202) |
|  | Indirect | 0.296 | 0.091 | .001 | (0.124\|0.480) | | 0.332 | 0.158 | .036 | (0.026\|0.648) | 0.298 | 0.038 | .001 | (0.225\|0.377) |
| Austria | Direct | 0.132 | 0.021 | <.001 | (0.091\|0.175) | | 0.313 | 0.023 | <.001 | (0.267\|0.357) | 0.050 | 0.010 | <.001 | (0.030\|0.070) |
|  | Indirect | 0.168 | 0.014 | <.001 | (0.143\|0.195) | | 0.074 | 0.015 | <.001 | (0.044\|0.104) | 0.064 | 0.005 | <.001 | (0.055\|0.073) |
| China | Direct | 0.504 | 0.072 | <.001 | (0.369\|0.649) | | 0.495 | 0.104 | <.001 | (0.302\|0.712) | 0.112 | 0.050 | .024 | (0.016\|0.211) |
|  | Indirect | 0.115 | 0.038 | .002 | (0.044\|0.192) | | 0.124 | 0.068 | .067 | (-0.014\|0.251) | 0.194 | 0.035 | <.001 | (0.133\|0.271) |
| Croatia | Direct | 0.344 | 0.069 | <.001 | (0.211\|0.483) | | 0.422 | 0.075 | <.001 | (0.274\|0.569) | 0.205 | 0.066 | .002 | (0.082\|0.343) |
|  | Indirect | 0.124 | 0.038 | .001 | (0.059\|0.207) | | 0.061 | 0.045 | .173 | (-0.029\|0.146) | 0.128 | 0.034 | <.001 | (0.067\|0.204) |
| Estonia | Direct | 0.374 | 0.332 | .259 | (.0079\|0.026) | | 0.134 | 0.158 | .398 | (-0.236\|0.386) | -0.022 | 0.075 | .774 | (-0.171\|0.124) |
|  | Indirect | 0.049 | 0.286 | .865 | (-0.416\|0.250) | | 0.190 | 0.119 | .110 | (0.022\|0.487) | 0.108 | 0.049 | .027 | (0.032\|0.230) |
| Finland | Direct | 0.171 | 0.032 | <.001 | (0.109\|0.236) | | 0.343 | 0.025 | <.001 | (0.296\|0.390) | 0.065 | 0.019 | .001 | (0.027\|0.102) |
|  | Indirect | 0.104 | 0.015 | <.001 | (0.076\|0.136) | | 0.039 | 0.012 | .002 | (0.016\|0.065) | 0.071 | 0.009 | <.001 | (0.054\|0.091) |
| Germany | Direct | 0.220 | 0.062 | <.001 | (0.103\|0.346) | | 0.391 | 0.063 | <.001 | (0.272\|0.519) | -0.007 | 0.034 | .831 | (-0.075\|0.058) |
|  | Indirect | 0.136 | 0.037 | <.001 | (0.062\|0.209) | | 0.058 | 0.042 | .171 | (-0.029\|0.136) | 0.065 | 0.016 | <.001 | (0.036\|0.099) |
| Iceland | Direct | 0.197 | 0.091 | .031 | (0.015\|.374) | | 0.270 | 0.075 | <.001 | (0.125\|0.415) | 0.042 | 0.039 | .275 | (-0.035\|0.119) |
|  | Indirect | 0.127 | 0.050 | .011 | (0.035\|0.237) | | 0.084 | 0.048 | .076 | (-0.007\|0.180) | 0.059 | 0.018 | .001 | (0.031\|0.101) |
| Japan | Direct | 0.331 | 0.151 | .028 | (0.092\|0.673) | | 0.226 | 0.054 | <.001 | (0.120\|0.333) | 0.100 | 0.037 | .006 | (0.031\|0.175) |
|  | Indirect | 0.047 | 0.108 | .664 | (-0.198\|0.213) | | 0.074 | 0.031 | .019 | (0.013\|0.137) | 0.072 | 0.018 | <.001 | (0.041\|0.111) |
| Kosovo | Direct | 0.268 | 0.051 | <.001 | (0.168\|0.369) | | 0.364 | 0.053 | <.001 | (0.261\|0.469) | 0.127 | 0.034 | <.001 | (0.062\|0.193) |
|  | Indirect | 0.203 | 0.031 | <.001 | (0.149\|0.269) | | 0.138 | 0.035 | <.001 | (0.073\|0.208) | 0.147 | 0.018 | <.001 | (0.115\|0.186) |
| Lithuania | Direct | 0.171 | 0.089 | .054 | (0.004\|0.352) | | 0.450 | 0.090 | <.001 | (0.276\|0.625) | 0.016 | 0.051 | .757 | (-0.080\|0.117) |
|  | Indirect | 0.123 | 0.049 | .012 | (0.034\|0.233) | | 0.007 | 0.054 | .902 | (-0.111\|0.103) | 0.075 | 0.025 | .003 | (0.034\|0.131) |
| Malta | Direct | 0.355 | 0.110 | .001 | (0.152\|0.578) | | 0.315 | 0.098 | .001 | (0.109\|0.498) | 0.050 | 0.067 | .458 | (-0.085\|0.181) |
|  | Indirect | 0.135 | 0.065 | .038 | (0.011\|0.265) | | 0.113 | 0.067 | .094 | (-0.010\|0.258) | 0.086 | 0.032 | .008 | (0.032\|0.161) |
| North Macedonia | Direct | 0.376 | 0.150 | .012 | (0.123\|0.691) | | 0.606 | 0.155 | <.001 | (0.326\|0.929) | 0.104 | 0.055 | .059 | (-0.012\|0.209) |
|  | Indirect | 0.177 | 0.083 | .032 | (0.035\|0.359) | | 0.097 | 0.083 | .245 | (-0.064\|0.261) | 0.129 | 0.034 | <.001 | (0.075\|0.211) |
| Poland | Direct | 0.225 | 0.087 | .010 | (0.056\|0.394) | | 0.446 | 0.066 | <.001 | (0.320\|0.577) | 0.072 | 0.038 | .055 | (0.001\|0.149) |
|  | Indirect | 0.080 | 0.057 | .161 | (-0.029\|0.191) | | -0.048 | 0.042 | .260 | (-0.134\|0.033) | 0.060 | 0.015 | <.001 | (0.035\|0.092) |
| Romania | Direct | 0.354 | 0.078 | <.001 | (0.205\|0.510) | | 0.266 | 0.079 | .001 | (0.113\|0.421) | 0.019 | 0.050 | .710 | (-0.079\|0.117) |
|  | Indirect | 0.110 | 0.045 | .015 | (0.023\|0.204) | | 0.134 | 0.050 | .008 | (0.035\|0.234) | 0.115 | 0.029 | <.001 | (0.066\|0.182) |
| Sweden | Direct | 0.476 | 0.037 | <.001 | (0.403\|0.548) | | 0.389 | 0.032 | <.001 | (0.326\|0.451) | 0.088 | 0.019 | <.001 | (0.050\|0.126) |
|  | Indirect | 0.035 | 0.021 | .094 | (-0.006\|0.075) | | 0.050 | 0.018 | .007 | (0.014\|0.087) | 0.089 | 0.010 | <.001 | (0.070\|0.109) |
| USA | Direct | -0.026 | 0.052 | .621 | (-0.127\|0.080) | | -0.027 | 0.036 | .453 | (-0.097\|0.046) | -0.023 | 0.026 | .380 | (-0.073\|0.027) |
|  | Indirect | 0.001 | 0.005 | .816 | (-0.005\|0.014) | | 0.001 | 0.004 | .772 | (-0.005\|0.012) | 0.000 | 0.002 | .904 | (-0.006\|0.002) |
| *Note.* CI = two-sided 5% confidence intervals | | | | | |  |  |  |  |  |  |  |  |  |

| **Table S25**  *Standardized path coefficients, standard errors, p-values, and two-sided 5 % confidence intervals for the multigroup mediation models predicting procrastination* | | | | | | | | | | | | | | |
| --- | --- | --- | --- | --- | --- | --- | --- | --- | --- | --- | --- | --- | --- | --- |
|  |  | Autonomy | | | | | Competence | | | | Social Relatedness | | | |
|  |  | Est. | *S.E.* | *p* | | CI | Est. | *S.E.* | *p* | CI | Est. | *S.E.* | *p* | CI |
| Albania | Direct | -0.267 | 0.148 | .072 | | (-0.593\|0.042) | -0.120 | 0.212 | .569 | (-0.575\|0.275) | 0.101 | 0.075 | .181 | (-0.050\|0.230) |
|  | Indirect | -0.326 | 0.132 | .013 | | (-0.550\|0.040) | -0.461 | 0.204 | .024 | (-0.572\|0.271) | -0.455 | 0.060 | <.001 | (-0.051\|0.242) |
| Austria | Direct | -0.073 | 0.025 | .004 | | (-0.154\|-0.029) | -0.382 | 0.026 | <.001 | (-0.539\|-0.410) | -0.057 | 0.015 | <.001 | (-0.090\|-0.028) |
|  | Indirect | -0.249 | 0.017 | <.001 | | (-0.123\|-0.023) | -0.086 | 0.019 | <.001 | (-0.431\|-0.329) | -0.095 | 0.007 | <.001 | (-0.086\|-0.027) |
| China | Direct | -0.178 | 0.091 | .052 | | (-0.473\|0.008) | -0.102 | 0.113 | .366 | (-0.461\|0.167) | -0.029 | 0.076 | .697 | (-0.195\|0.125) |
|  | Indirect | 0.028 | 0.050 | .581 | | (-0.353\|0.007) | 0.008 | 0.074 | .910 | (-0.321\|0.116) | -0.028 | 0.048 | .564 | (-0.183\|0.115) |
| Croatia | Direct | -0.099 | 0.077 | .199 | | (-0.338\|0.095) | -0.168 | 0.086 | .050 | (-0.421\|0.000) | 0.043 | 0.086 | .617 | (-0.144\|0.251) |
|  | Indirect | -0.209 | 0.049 | <.001 | | (-0.236\|0.072) | -0.210 | 0.057 | <.001 | (-0.342\|0.000) | -0.247 | 0.049 | <.001 | (-0.122\|0.215) |
| Estonia | Direct | -0.254 | 0.306 | .407 | | (-1.060\|0.115) | -0.328 | 0.184 | .075 | (-0.868\|0.027) | 0.333 | 0.122 | .006 | (0.092\|0.619) |
|  | Indirect | -0.323 | 0.256 | .207 | | (-0.859\|0.091) | -0.274 | 0.129 | .034 | (-0.678\|0.028) | -0.249 | 0.099 | .012 | (0.080\|0.557) |
| Finland | Direct | -0.147 | 0.036 | <.001 | | (-0.279\|-0.101) | -0.480 | 0.031 | <.001 | (-0.579\|-0.443) | -0.102 | 0.031 | .001 | (-0.157\|-0.040) |
|  | Indirect | -0.130 | 0.017 | <.001 | | (-0.218\|-0.079) | -0.047 | 0.017 | .007 | (-0.541\|-0.418) | -0.109 | 0.014 | <.001 | (-0.163\|-0.042) |
| Germany | Direct | -0.264 | 0.064 | <.001 | | (-0.545\|-0.182) | -0.408 | 0.067 | <.001 | (-0.775\|-0.394) | -0.017 | 0.042 | .694 | (-0.123\|0.084) |
|  | Indirect | -0.155 | 0.040 | <.001 | | (-0.386\|-0.133) | -0.083 | 0.048 | .081 | (-0.542\|-0.281) | -0.085 | 0.020 | <.001 | (-0.099\|0.066) |
| Iceland | Direct | -0.169 | 0.097 | .082 | | (-0.530\|0.030) | -0.359 | 0.083 | <.001 | (-0.674\|-0.256) | 0.009 | 0.061 | .886 | (-0.112\|0.125) |
|  | Indirect | -0.150 | 0.053 | .005 | | (-0.357\|0.021) | -0.081 | 0.052 | .122 | (-0.515\|-0.196) | -0.099 | 0.025 | <.001 | (-0.111\|0.127) |
| Japan | Direct | -0.288 | 0.134 | .032 | | (-0.969\|-0.088) | -0.221 | 0.065 | .001 | (-0.433\|-0.112) | 0.098 | 0.050 | .051 | (-0.001\|0.217) |
|  | Indirect | -0.036 | 0.099 | .715 | | (-0.588\|-0.065) | -0.082 | 0.038 | .029 | (-0.347\|-0.095) | -0.127 | 0.025 | <.001 | (0.004\|0.199) |
| Kosovo | Direct | -0.116 | 0.050 | .020 | | (-0.328\|-0.028) | -0.214 | 0.055 | <.001 | (-0.426\|-0.139) | -0.016 | 0.043 | .717 | (-0.115\|0.084) |
|  | Indirect | -0.143 | 0.028 | <.001 | | (-0.218\|-0-020) | -0.114 | 0.037 | .002 | (-0.319\|-0.107) | -0.135 | 0.020 | <.001 | (-0.098\|0.070) |
| Lithuania | Direct | -0.248 | 0.089 | .005 | | (-0.618\|-0.095) | -0.604 | 0.091 | <.001 | (-0.989\|-0.518) | -0.104 | 0.074 | .162 | (-0.272\|0.044) |
|  | Indirect | -0.077 | 0.044 | .081 | | (-0.420\|-0.077) | 0.057 | 0.060 | .339 | (-0.802\|-0.443) | -0.081 | 0.029 | .005 | (-0.260\|0.036) |
| Malta | Direct | -0.358 | 0.132 | .007 | | (-0.871\|-0.136) | -0.497 | 0.117 | <.001 | (-0.819\|-0.288) | -0.031 | 0.085 | .718 | (-0.234\|0.149) |
|  | Indirect | -0.125 | 0.075 | .097 | | (-0.129\|0.138) | -0.049 | 0.076 | .517 | (-0.703\|-0.250) | -0.104 | 0.039 | .008 | (-0.203\|0.130) |
| North Macedonia | Direct | -0.188 | 0.127 | .139 | | (-0.631\|0.091) | -0.550 | 0.109 | <.001 | (-1.190\|-0.510) | -0.110 | 0.081 | .172 | (-0.256\|0.057) |
|  | Indirect | -0.272 | 0.085 | .001 | | (-0.452\|0.049) | -0.119 | 0.078 | .126 | (-0.814\|-0.373) | -0.205 | 0.048 | <.001 | (-0.260\|0.054) |
| Poland | Direct | -0.229 | 0.088 | .009 | | (-0.597\|-0.075) | -0.505 | 0.070 | <.001 | (-0.865\|-0.478) | -0.036 | 0.052 | .490 | (-0.159\|0.078) |
|  | Indirect | -0.103 | 0.057 | .070 | | (-0.401\|-0.056) | 0.038 | 0.049 | .441 | (-0.637\|-0.364) | -0.097 | 0.021 | <.001 | (-0.139\|0.065) |
| Romania | Direct | -0.230 | 0.100 | .021 | | (-0.472\|-0.017) | -0.289 | 0.096 | .003 | (-0.493\|-0.082) | -0.165 | 0.071 | .019 | (-0.287\|-0.010) |
|  | Indirect | -0.119 | 0.059 | .043 | | (-0.444\|-0.042) | -0.092 | 0.065 | .158 | (-0.495\|-0.111) | -0.094 | 0.028 | .001 | (-0.314\|-0.039) |
| Sweden | Direct | -0.464 | 0.038 | <.001 | | (-0.769\|-0.545) | -0.394 | 0.037 | <.001 | (-0.603\|-0.412) | -0.110 | 0.029 | <.001 | (-0.170\|-0.051) |
|  | Indirect | -0.045 | 0.024 | .060 | | (-0.538\|-0.385) | -0.071 | 0.023 | .002 | (-0.467\|-0.321) | -0.132 | 0.014 | <.001 | (-0.167\|-0.049) |
| USA | Direct | -0.021 | 0.058 | .715 | | (-0.146\|0.108) | -0.021 | 0.053 | .689 | (-0.097\|0.068) | -0.013 | 0.048 | .791 | (-0.067\|0.051) |
|  | Indirect | 0.001 | 0.005 | .807 | | (-0.133\|0.093) | 0.002 | 0.006 | .777 | (-0.124\|0.086) | 0.000 | 0.003 | .882 | (-0.104\|0.081) |
| *Note.* CI = two-sided 5% confidence intervals | | | | |  |  |  |  |  |  |  |  |  |  |

| **Table S26**  *Standardized path coefficients, standard errors, p-values, and two-sided 5 % confidence intervals for the multigroup mediation models predicting persistence* | | | | | | | | | | | | | | |
| --- | --- | --- | --- | --- | --- | --- | --- | --- | --- | --- | --- | --- | --- | --- |
|  |  | Autonomy | | | | | Competence | | | | Social Relatedness | | | |
|  |  | Est. | *S.E.* | *p* | | CI | Est. | *S.E.* | *p* | CI | Est. | *S.E.* | *p* | CI |
| Albania | Direct | 0.353 | 0.140 | .012 | | (0.060\|0.489) | 0.285 | 0.227 | .210 | (-0.126\|0.539) | 0.141 | 0.076 | .063 | (-0.003\|0.202) |
|  | Indirect | 0.387 | 0.120 | .001 | | (0.077\|0.620) | 0.455 | 0.218 | .037 | (-0.167\|0.733) | 0.440 | 0.056 | <.001 | (-0.004\|0.293) |
| Austria | Direct | 0.179 | 0.028 | <.001 | | (0.091\|0.175) | 0.421 | 0.029 | <.001 | (0.267\|0.357) | 0.082 | 0.017 | <.001 | (0.030\|0.070) |
|  | Indirect | 0.228 | 0.018 | <.001 | | (0.123\|0.234) | 0.099 | 0.020 | <.001 | (0.362\|0.476) | 0.104 | 0.007 | <.001 | (0.049\|0.114) |
| China | Direct | 0.562 | 0.076 | <.001 | | (0.328\|0.649) | 0.511 | 0.100 | <.001 | (0.302\|0.712) | 0.157 | 0.067 | .019 | (0.016\|0.211) |
|  | Indirect | 0.128 | 0.041 | .002 | | (0.415\|0.712) | 0.129 | 0.069 | .063 | (0.324\|0.723) | 0.271 | 0.046 | <.001 | (0.031\|0.292) |
| Croatia | Direct | 0.437 | 0.085 | <.001 | | (0.211\|0.483) | 0.601 | 0.098 | <.001 | (0.274\|0.569) | 0.305 | 0.093 | .001 | (0.082\|0.343) |
|  | Indirect | 0.158 | 0.046 | .001 | | (0.266\|0.604) | 0.087 | 0.065 | .176 | (0.405\|0.791) | 0.190 | 0.049 | <.001 | (0.127\|0.490) |
| Estonia | Direct | 0.722 | 0.572 | .207 | | (0.079\|1.026) | 0.232 | 0.278 | .405 | (-0.236\|0.386) | -0.042 | 0.140 | .762 | (-0.171\|0.124) |
|  | Indirect | 0.094 | 0.501 | .852 | | (0.140\|1.801) | 0.329 | 0.182 | .070 | (-0.366\|0.745) | 0.211 | 0.091 | .020 | (-0.312\|0.246) |
| Finland | Direct | 0.232 | 0.042 | <.001 | | (0.109\|0.236) | 0.551 | 0.036 | <.001 | (0.296\|0.390) | 0.118 | 0.035 | .001 | (0.027\|0.102) |
|  | Indirect | 0.141 | 0.020 | <.001 | | (0.151\|0.314) | 0.063 | 0.020 | .001 | (0.478\|0.618) | 0.129 | 0.016 | <.001 | (0.049\|0.184) |
| Germany | Direct | 0.307 | 0.083 | <.001 | | (0.103\|0.346) | 0.530 | 0.081 | <.001 | (0.272\|0.519) | -0.011 | 0.053 | .830 | (-0.075\|0.058) |
|  | Indirect | 0.190 | 0.052 | <.001 | | (0.143\|0.471) | 0.078 | 0.057 | .169 | (0.373\|0.696) | 0.102 | 0.025 | <.001 | (-0.116\|0.091) |
| Iceland | Direct | 0.245 | 0.110 | .026 | | (0.015\|0.374) | 0.372 | 0.101 | <.001 | (0.125\|0.415) | 0.078 | 0.072 | .277 | (-0.035\|0.119) |
|  | Indirect | 0.158 | 0.062 | .011 | | (0.017\|0.457) | 0.116 | 0.066 | .077 | (0.170\|0.564) | 0.108 | 0.032 | .001 | (-0.064\|0.214) |
| Japan | Direct | 0.418 | 0.162 | .010 | | (0.092\|0.673) | 0.347 | 0.078 | <.001 | (0.120\|0.333) | 0.179 | 0.059 | .002 | (0.031\|0.175) |
|  | Indirect | 0.059 | 0.121 | .623 | | (0.166\|0.857) | 0.114 | 0.045 | .011 | (0.222\|0.532) | 0.129 | 0.029 | <.001 | (0.082\|0.313) |
| Kosovo | Direct | 0.285 | 0.052 | <.001 | | (0.168\|0.369) | 0.435 | 0.063 | <.001 | (0.261\|0.469) | 0.173 | 0.044 | <.001 | (0.062\|0.193) |
|  | Indirect | 0.216 | 0.032 | <.001 | | (0.194\|0.398) | 0.164 | 0.040 | <.001 | (0.331\|0.576) | 0.201 | 0.025 | <.001 | (0.092\|0.263) |
| Lithuania | Direct | 0.203 | 0.100 | .042 | | (0.004\|0.352) | 0.615 | 0.115 | <.001 | (0.276\|0.625) | 0.026 | 0.083 | .754 | (-0.080\|0.117) |
|  | Indirect | 0.146 | 0.057 | .011 | | (0.009\|0.402) | 0.009 | 0.073 | .901 | (0.396\|0.848) | 0.124 | 0.040 | .002 | (-0.132\|0.192) |
| Malta | Direct | 0.398 | 0.119 | .001 | | (0.152\|0.578) | 0.397 | 0.122 | .001 | (0.109\|0.498) | 0.066 | 0.090 | .458 | (-0.085\|0.181) |
|  | Indirect | 0.152 | 0.072 | .035 | | (-0.118\|0.176) | 0.142 | 0.083 | .088 | (0.129\|0.614) | 0.114 | 0.041 | .006 | (-0.109\|0.244) |
| North Macedonia | Direct | 0.420 | 0.148 | .004 | | (0.123\|0.691) | 0.643 | 0.142 | <.001 | (0.326\|0.929) | 0.168 | 0.087 | .054 | (-0.012\|0.209) |
|  | Indirect | 0.198 | 0.089 | .027 | | (0.144\|0.715) | 0.103 | 0.088 | .242 | (0.378\|0.931) | 0.208 | 0.050 | <.001 | (-0.015\|0.334) |
| Poland | Direct | 0.286 | 0.106 | .007 | | (0.056\|0.394) | 0.623 | 0.089 | <.001 | (0.320\|0.577) | 0.120 | 0.062 | .051 | (0.001\|0.149) |
|  | Indirect | 0.102 | 0.072 | .157 | | (0.076\|0.489) | -0.067 | 0.059 | .257 | (0.448\|0.799) | 0.100 | 0.024 | <.001 | (0.004\|0.242) |
| Romania | Direct | 0.477 | 0.095 | <.001 | | (0.205\|0.510) | 0.379 | 0.102 | <.001 | (0.113\|0.421) | 0.029 | 0.074 | .699 | (-0.079\|0.117) |
|  | Indirect | 0.149 | 0.060 | .013 | | (0.292\|0.667) | 0.191 | 0.071 | .007 | (0.179\|0.586) | 0.178 | 0.043 | <.001 | (-0.112\|0.179) |
| Sweden | Direct | 0.582 | 0.041 | <.001 | | (0.403\|0.548) | 0.520 | 0.040 | <.001 | (0.326\|0.451) | 0.149 | 0.033 | <.001 | (0.050\|0.126) |
|  | Indirect | 0.042 | 0.025 | .096 | | (0.500\|0.659) | 0.067 | 0.025 | .007 | (0.438\|0.596) | 0.152 | 0.016 | <.001 | (0.084\|0.213) |
| USA | Direct | -0.031 | 0.060 | .606 | | (-0.127\|0.080) | -0.046 | 0.059 | .436 | (-0.097\|0.046) | -0.048 | 0.053 | .365 | (-0.073\|0.027) |
|  | Indirect | 0.001 | 0.005 | .810 | | (-0.146\|0.089) | 0.002 | 0.006 | .763 | (-0.161\|0.071) | 0.000 | 0.003 | .900 | (-0.154\|0.053) |
| *Note.* CI = two-sided 5% confidence intervals | | | | |  |  |  |  |  |  |  |  |  |  |

| **Table S27**  *Standardized path coefficients of direct, and indirect effects on procrastination in the multigroup models with fixed paths* | | | | | | | | | | | | | | |
| --- | --- | --- | --- | --- | --- | --- | --- | --- | --- | --- | --- | --- | --- | --- |
| Country |  | Autonomy | | | | Competence | | | | Social Relatedness | | | |  |
|  |  | Est. | *S.E.* | *p* | CI | Est. | *S.E.* | *p* | CI | Est. | *S.E.* | *p* | CI |  |
| Albania | Direct | -0.280 | 0.021 | <.001 | (-0.323\|-0.237) | -0.461 | 0.023 | <.001 | (-0.511\|-0.420) | -0.073 | 0.013 | <.001 | (-0.098\|-0.049) |  |
|  | Indirect | -0.213 | 0.015 | <.001 | (-0.243\|-0.187) | -0.116 | 0.012 | <.001 | (-0.143\|-0.094) | -0.142 | 0.009 | <.001 | (-0.161\|-0.127) |  |
| Austria | Direct | -0.215 | 0.014 | <.001 | (-0.243\|-0.185) | -0.362 | 0.014 | <.001 | (-0.388\|-0.334) | -0.059 | 0.010 | <.001 | (-0.077\|-0.040) |  |
|  | Indirect | -0.163 | 0.009 | <.001 | (-0.181\|-0.145) | -0.091 | 0.009 | <.001 | (-0.109\|-0.074) | -0.115 | 0.004 | <.001 | (-0.123\|-0.106) |  |
| China | Direct | -0.184 | 0.015 | <.001 | (-0.213\|-0.156) | -0.288 | 0.017 | <.001 | (-0.322\|-0.257) | -0.054 | 0.009 | <.001 | (-0.073\|-0.037) |  |
|  | Indirect | -0.140 | 0.010 | <.001 | (-0.159\|-0.121) | -0.073 | 0.008 | <.001 | (-0.089\|-0.058) | -0.105 | 0.007 | <.001 | (-0.119\|-0.093) |  |
| Croatia | Direct | -0.196 | 0.016 | <.001 | (-0.235\|-0.163) | -0.357 | 0.022 | <.001 | (-0.403\|-0.314) | -0.056 | 0.010 | <.001 | (-0.076\|-0.037) |  |
|  | Indirect | -0.149 | 0.011 | <.001 | (-0.175\|-0.126) | -0.090 | 0.010 | <.001 | (-0.111\|-0.072) | -0.109 | 0.008 | <.001 | (-0.126\|-0.094) |  |
| Estonia | Direct | -0.268 | 0.031 | <.001 | (-0.335\|-0.213) | -0.404 | 0.041 | <.001 | (-0.491\|-0.326) | -0.061 | 0.012 | <.001 | (-0.086\|-0.041) |  |
|  | Indirect | -0.204 | 0.023 | <.001 | (-0.250\|-0.165) | -0.102 | 0.014 | <.001 | (-0.131\|-0.077) | -0.119 | 0.014 | <.001 | (-0.149\|-0.094) |  |
| Finland | Direct | -0.192 | 0.013 | <.001 | (-0.219\|-0.165) | -0.402 | 0.017 | <.001 | (-0.436\|-0.369) | -0.064 | 0.011 | <.001 | (-0.084\|-0.043) |  |
|  | Indirect | -0.146 | 0.008 | <.001 | (-0.163\|-0.129) | -0.101 | 0.010 | <.001 | (-0.122\|-0.083) | -0.124 | 0.005 | <.001 | (-0.134\|-0.114) |  |
| Germany | Direct | -0.199 | 0.015 | <.001 | (-0.231\|-0.169) | -0.329 | 0.016 | <.001 | (-0.362\|-0.299) | -0.050 | 0.008 | <.001 | (-0.066\|-0.034) |  |
|  | Indirect | -0.151 | 0.010 | <.001 | (-0.172\|-0.133) | -0.083 | 0.008 | <.001 | (-0.100\|-0.067) | -0.097 | 0.005 | <.001 | (-0.107\|-0.087) |  |
| Iceland | Direct | -0.185 | 0.016 | <.001 | (-0.217\|-0.154) | -0.338 | 0.021 | <.001 | (-0.381\|-0.298) | -0.060 | 0.010 | <.001 | (-0.080\|-0.041) |  |
|  | Indirect | -0.140 | 0.011 | <.001 | (-0.162\|-0.120) | -0.085 | 0.009 | <.001 | (-0.104\|-0.069) | -0.117 | 0.007 | <.001 | (-0.130\|-0.104) |  |
| Japan | Direct | -0.176 | 0.014 | <.001 | (-0.207\|-0.148) | -0.320 | 0.019 | <.001 | (-0.359\|-0.288) | -0.055 | 0.009 | <.001 | (-0.074\|-0.039) |  |
|  | Indirect | -0.134 | 0.009 | <.001 | (-0.155\|-0.116) | -0.080 | 0.009 | <.001 | (-0.099\|-0.065) | -0.107 | 0.007 | <.001 | (-0.122\|-0.096) |  |
| Kosovo | Direct | -0.176 | 0.013 | <.001 | (-0.203\|-0.151) | -0.324 | 0.015 | <.001 | (-0.355\|-0.296) | -0.053 | 0.009 | <.001 | (-0.070\|-0.036) |  |
|  | Indirect | -0.134 | 0.008 | <.001 | (-0.152\|-0.118) | -0.082 | 0.008 | <.001 | (-0.098\|-0.066) | -0.102 | 0.005 | <.001 | (-0.113\|-0.093) |  |
| Lithuania | Direct | -0.179 | 0.016 | <.001 | (-0.218\|-0.152) | -0.358 | 0.024 | <.001 | (-0.418\|-0.321) | -0.059 | 0.010 | <.001 | (-0.080\|-0.041) |  |
|  | Indirect | -0.136 | 0.011 | <.001 | (-0.161\|-0.119) | -0.090 | 0.010 | <.001 | (-0.113\|-0.075) | -0.114 | 0.007 | <.001 | (-0.132\|-0.102) |  |
| Malta | Direct | -0.205 | 0.020 | <.001 | (-0.245\|-0.169) | -0.384 | 0.027 | <.001 | (-0.438\|-0.332) | -0.055 | 0.010 | <.001 | (-0.074\|-0.037) |  |
|  | Indirect | -0.156 | 0.014 | <.001 | (-0.183\|-0.132) | -0.097 | 0.011 | <.001 | (-0.118\|-0.076) | -0.106 | 0.008 | <.001 | (-0.123\|-0.090) |  |
| North Macedonia | Direct | -0.211 | 0.021 | <.001 | (-0.262\|-0.174) | -0.358 | 0.031 | <.001 | (-0.436\|-0.310) | -0.069 | 0.012 | <.001 | (-0.095\|-0.047) |  |
|  | Indirect | -0.160 | 0.014 | <.001 | (-0.197\|-0.136) | -0.090 | 0.011 | <.001 | (-0.117\|-0.073) | -0.133 | 0.010 | <.001 | (-0.159\|-0.117) |  |
| Poland | Direct | -0.185 | 0.014 | <.001 | (-0.216\|-0.159) | -0.335 | 0.017 | <.001 | (-0.374\|-0.305) | -0.054 | 0.009 | <.001 | (-0.072\|-0.037) |  |
|  | Indirect | -0.141 | 0.009 | <.001 | (-0.162\|-0.124) | -0.084 | 0.009 | <.001 | (-0.103\|-0.069) | -0.104 | 0.006 | <.001 | (-0.117\|-0.095) |  |
| Romania | Direct | -0.236 | 0.020 | <.001 | (-0.291\|-0.204) | -0.399 | 0.027 | <.001 | (-0.470\|-0.362) | -0.064 | 0.011 | <.001 | (-0.094\|-0.047) |  |
|  | Indirect | -0.179 | 0.013 | <.001 | (-0.218\|-0.158) | -0.100 | 0.011 | <.001 | (-0.129\|-0.083) | -0.125 | 0.011 | <.001 | (-0.160\|-0.113) |  |
| Sweden | Direct | -0.199 | 0.014 | <.001 | (-0.227\|-0.168) | -0.348 | 0.015 | <.001 | (-0.378\|-0.318) | -0.061 | 0.010 | <.001 | (-0.080\|-0.041) |  |
|  | Indirect | -0.151 | 0.008 | <.001 | (-0.167\|-0.133) | -0.087 | 0.009 | <.001 | (-0.105\|-0.071) | -0.118 | 0.005 | <.001 | (-0.127\|-0.107) |  |
| USA | Direct | -0.188 | 0.015 | <.001 | (-0.230\|-0.160) | -0.389 | 0.020 | <.001 | (-0.431\|-0.358) | -0.080 | 0.013 | <.001 | (-0.107\|-0.055) |  |
|  | Indirect | -0.143 | 0.010 | <.001 | (-0.172\|-0.123) | -0.098 | 0.011 | <.001 | (-0.123\|-0.080) | -0.155 | 0.009 | <.001 | (-0.174\|-0.141) |  |

| **Table S28**  *Standardized path coefficients of direct, and indirect effects on persistence in the multigroup models with fixed paths* | | | | | | | | | | | | | |
| --- | --- | --- | --- | --- | --- | --- | --- | --- | --- | --- | --- | --- | --- |
| Country |  | Autonomy | | | | Competence | | | | Social Relatedness | | | |
|  |  | Est. | *S.E.* | *p* | CI | Est. | *S.E.* | *p* | CI | Est. | *S.E.* | *p* | CI |
| Albania | Direct | 0.381 | 0.026 | <.001 | (0.330\|0.436) | 0.512 | 0.029 | <.001 | (0.459\|0.572) | 0.115 | 0.014 | <.001 | (0.089\|0.144) |
|  | Indirect | 0.186 | 0.014 | <.001 | (0.160\|0.218) | 0.117 | 0.012 | <.001 | (0.094\|0.142) | 0.143 | 0.010 | <.001 | (0.126\|0.166) |
| Austria | Direct | 0.325 | 0.015 | <.001 | (0.291\|0.357) | 0.437 | 0.015 | <.001 | (0.405\|0.466) | 0.105 | 0.011 | <.001 | (0.083\|0.125) |
|  | Indirect | 0.159 | 0.009 | <.001 | (0.140\|0.179) | 0.100 | 0.010 | <.001 | (0.081\|0.118) | 0.130 | 0.005 | <.001 | (0.121\|0.140) |
| China | Direct | 0.300 | 0.022 | <.001 | (0.258\|0.355) | 0.373 | 0.026 | <.001 | (0.330\|0.434) | 0.098 | 0.011 | <.001 | (0.078\|0.124) |
|  | Indirect | 0.146 | 0.011 | <.001 | (0.126\|0.172) | 0.085 | 0.009 | <.001 | (0.069\|0.107) | 0.122 | 0.008 | <.001 | (0.109\|0.145) |
| Croatia | Direct | 0.327 | 0.027 | <.001 | (0.274\|0.386) | 0.487 | 0.033 | <.001 | (0.428\|0.555) | 0.105 | 0.013 | <.001 | (0.080\|0.132) |
|  | Indirect | 0.160 | 0.014 | <.001 | (0.133\|0.190) | 0.111 | 0.012 | <.001 | (0.088\|0.136) | 0.131 | 0.011 | <.001 | (0.112\|0.153) |
| Estonia | Direct | 0.492 | 0.073 | <.001 | (0.384\|0.704) | 0.550 | 0.091 | <.001 | (0.423\|0.781) | 0.125 | 0.026 | <.001 | (0.089\|0.197) |
|  | Indirect | 0.240 | 0.036 | <.001 | (0.186\|0.342) | 0.125 | 0.024 | <.001 | (0.91\|0.187) | 0.156 | 0.030 | <.001 | (0.117\|0.243) |
| Finland | Direct | 0.301 | 0.016 | <.001 | (0.267\|0.336) | 0.500 | 0.021 | <.001 | (0.459\|0.542) | 0.116 | 0.012 | <.001 | (0.092\|0.140) |
|  | Indirect | 0.147 | 0.009 | <.001 | (0.128\|0.168) | 0.114 | 0.011 | <.001 | (0.093\|0.136) | 0.145 | 0.007 | <.001 | (0.132\|0.158) |
| Germany | Direct | 0.343 | 0.022 | <.001 | (0.299\|0.392) | 0.460 | 0.026 | <.001 | (0.415\|0.516) | 0.101 | 0.011 | <.001 | (0.079\|0.124) |
|  | Indirect | 0.168 | 0.012 | <.001 | (0.144\|0.196) | 0.105 | 0.011 | <.001 | (0.085\|0.128) | 0.125 | 0.008 | <.001 | (0.112\|0.141) |
| Iceland | Direct | 0.301 | 0.026 | <.001 | (0.249\|0.357) | 0.437 | 0.033 | <.001 | (0.375\|0.501) | 0.113 | 0.014 | <.001 | (0.087\|0.143) |
|  | Indirect | 0.147 | 0.013 | <.001 | (0.123\|0.177) | 0.100 | 0.012 | <.001 | (0.079\|0.124) | 0.141 | 0.011 | <.001 | (0.120\|0.163) |
| Japan | Direct | 0.315 | 0.025 | <.001 | (0.291\|0.453) | 0.450 | 0.033 | <.001 | (0.417\|0.568) | 0.116 | 0.014 | <.001 | (0.099\|0.154) |
|  | Indirect | 0.154 | 0.013 | <.001 | (0.141\|0.214) | 0.103 | 0.011 | <.001 | (0.088\|0.139) | 0.144 | 0.012 | <.001 | (0.133\|0.179) |
| Kosovo | Direct | 0.276 | 0.016 | <.001 | (0.248\|0.332) | 0.412 | 0.025 | <.001 | (0.381\|0.469) | 0.095 | 0.010 | <.001 | (0.079\|0.122) |
|  | Indirect | 0.135 | 0.009 | <.001 | (0.121\|0.161) | 0.094 | 0.010 | <.001 | (0.080\|0.120) | 0.118 | 0.007 | <.001 | (0.109\|0.139) |
| Lithuania | Direct | 0.277 | 0.024 | <.001 | (0.233\|0.332) | 0.447 | 0.033 | <.001 | (0.392\|0.524) | 0.105 | 0.013 | <.001 | (0.082\|0.132) |
|  | Indirect | 0.135 | 0.012 | <.001 | (0.114\|0.165) | 0.102 | 0.011 | <.001 | (0.082\|0.127) | 0.131 | 0.010 | <.001 | (0.114\|0.154) |
| Malta | Direct | 0.289 | 0.027 | <.001 | (0.239\|0.350) | 0.415 | 0.035 | <.001 | (0.352\|0.489) | 0.088 | 0.012 | <.001 | (0.067\|0.112) |
|  | Indirect | 0.141 | 0.014 | <.001 | (0.116\|0.171) | 0.095 | 0.012 | <.001 | (0.074\|0.121) | 0.110 | 0.010 | <.001 | (0.091\|0.131) |
| North Macedonia | Direct | 0.303 | 0.030 | <.001 | (0.252\|0.372) | 0.410 | 0.037 | <.001 | (0.348\|0.496) | 0.113 | 0.015 | <.001 | (0.086\|0.147) |
|  | Indirect | 0.148 | 0.015 | <.001 | (0.123\|0.184) | 0.094 | 0.012 | <.001 | (0.074\|0.121) | 0.141 | 0.013 | <.001 | (0.120\|0.170) |
| Poland | Direct | 0.304 | 0.021 | <.001 | (0.265\|0.350) | 0.444 | 0.027 | <.001 | (0.395\|0.501) | 0.103 | 0.012 | <.001 | (0.081\|0.128) |
|  | Indirect | 0.149 | 0.011 | <.001 | (0.128\|0.175) | 0.101 | 0.011 | <.001 | (0.082\|0.124) | 0.128 | 0.009 | <.001 | (0.113\|0.147) |
| Romania | Direct | 0.343 | 0.027 | <.001 | (0.291\|0.416) | 0.459 | 0.032 | <.001 | (0.409\|0.541) | 0.104 | 0.014 | <.001 | (0.083\|0.139) |
|  | Indirect | 0.168 | 0.014 | <.001 | (0.142\|0.207) | 0.105 | 0.012 | <.001 | (0.085\|0.134) | 0.130 | 0.012 | <.001 | (0.111\|0.163) |
| Sweden | Direct | 0.316 | 0.017 | <.001 | (0.278\|0.354) | 0.441 | 0.019 | <.001 | (0.401\|0.478) | 0.110 | 0.012 | <.001 | (0.086\|0.132) |
|  | Indirect | 0.155 | 0.009 | <.001 | (0.136\|0.175) | 0.101 | 0.010 | <.001 | (0.082\|0.120) | 0.137 | 0.006 | <.001 | (0.124\|0.149) |
| USA | Direct | 0.226 | 0.016 | <.001 | (0.197\|0.277) | 0.366 | 0.019 | <.001 | (0.333\|0.408) | 0.110 | 0.012 | <.001 | (0.090\|0.138) |
|  | Indirect | 0.110 | 0.009 | <.001 | (0.094\|0.136) | 0.084 | 0.009 | <.001 | (0.068\|0.103) | 0.137 | 0.008 | <.001 | (0.125\|0.158) |

| **Table S29**  *Explained variance in % per model, outcome variable, and country for procrastination* | | | | | | |
| --- | --- | --- | --- | --- | --- | --- |
| Country | Autonomy | | Competence | | Social relatedness | |
|  | Multigroup | Fixed paths | Multigroup | Fixed paths | Multigroup | Fixed paths |
| Albania | 39.0 | 26.3 | 36.8 | 15.2 | 38.0 | 34.4 |
| Austria | 16.4 | 17.4 | 16.5 | 15.5 | 22.4 | 21.4 |
| China | 2.5 | 13.2 | 0.5 | 9.0 | 1.0 | 13.9 |
| Croatia | 20.5 | 17.1 | 19.5 | 13.7 | 21.2 | 21.9 |
| Estonia | 44.4 | 27.7 | 48.1 | 19.3 | 44.2 | 27.2 |
| Finland | 13.9 | 15.4 | 13.8 | 13.4 | 29.2 | 27.3 |
| Germany | 23.0 | 17.8 | 18.6 | 15.0 | 27.1 | 20.3 |
| Iceland | 16.6 | 17.0 | 15.2 | 18.2 | 21.8 | 20.7 |
| Japan | 13.7 | 14.2 | 10.7 | 11.3 | 13.2 | 19.4 |
| Kosovo | 11.0 | 13.0 | 10.0 | 11.0 | 12.6 | 17.5 |
| Lithuania | 17.0 | 17.8 | 12.1 | 14.6 | 33.8 | 24.6 |
| Malta | 25.9 | 16.5 | 18.5 | 13.7 | 30.2 | 24.1 |
| North Macedonia | 36.4 | 21.5 | 34.1 | 17.9 | 51.9 | 26.3 |
| Poland | 14.1 | 15.4 | 11.3 | 13.6 | 23.6 | 20.1 |
| Romania | 15.5 | 21.5 | 15.2 | 17.0 | 17.0 | 26.8 |
| Sweden | 27.0 | 17.6 | 16.0 | 16.3 | 23.5 | 21.3 |
| USA | 2.2 | 21.8 | 2.1 | 23.0 | 2.1 | 27.4 |
| Average | 19.9 | 18.3 | 17.6 | 15.2 | 24.3 | 23.2 |

| **Table S30**  *Explained variance in % per model, outcome variable, and country for persistence* | | | | | | |
| --- | --- | --- | --- | --- | --- | --- |
| Country | Autonomy | | Competence | | Social Relatedness | |
|  | Multigroup | Fixed paths | Multigroup | Fixed paths | Multigroup | Fixed paths |
| Austria | 21.8 | 26.5 | 27.9 | 30.0 | 20.8 | 22.0 |
| Albania | 59.6 | 34.1 | 58.1 | 40.0 | 56.5 | 19.1 |
| China | 51.2 | 24.7 | 44.3 | 24.6 | 33.6 | 17.2 |
| Croatia | 41.4 | 28.5 | 48.7 | 37.5 | 33.4 | 20.4 |
| Estonia | 69.9 | 63.2 | 52.1 | 55.9 | 50.6 | 48.3 |
| Finland | 20.7 | 23.7 | 39.1 | 39.4 | 17.8 | 19.2 |
| Germany | 29.3 | 30.1 | 37.8 | 33.7 | 23.8 | 23.7 |
| Iceland | 22.1 | 25.9 | 26.4 | 31.0 | 19.4 | 26.1 |
| Japan | 24.6 | 25.8 | 24.7 | 32.8 | 19.3 | 19.8 |
| Kosovo | 35.1 | 21.0 | 40.0 | 27.6 | 31.4 | 16.8 |
| Lithuania | 19.5 | 21.9 | 40.2 | 32.5 | 15.7 | 17.7 |
| Malta | 33.7 | 21.2 | 31.0 | 26.8 | 23.1 | 15.6 |
| North Macedonia | 44.2 | 23.4 | 57.3 | 26.8 | 35.9 | 18.3 |
| Poland | 16.7 | 24.1 | 31.6 | 31.1 | 13.9 | 20.4 |
| Romania | 42.5 | 29.5 | 37.7 | 33.8 | 29.3 | 19.7 |
| Sweden | 39.7 | 27.2 | 35.7 | 31.6 | 21.4 | 22.6 |
| USA | 0.9 | 18.2 | 1.0 | 23.5 | 1.0 | 20.0 |
| Average | 33.7 | 27.6 | 37.3 | 32.9 | 26.3 | 21.6 |
